# A systematic review and meta-analyses of risk factors associated with lameness in dairy cows

**DOI:** 10.1186/s12917-019-2095-2

**Published:** 2019-10-16

**Authors:** Andreas W. Oehm, Gabriela Knubben-Schweizer, Anna Rieger, Alexander Stoll, Sonja Hartnack

**Affiliations:** 1 Clinic for Ruminants with Ambulatory and Herd Health Services , Ludwig-Maximilians-Universität München, Sonnenstrasse 16, 85764 Oberschleissheim, Germany; 2 Section of Epidemiology, Vetsuisse Faculty, University of Zurich, Winterthurerstrasse 270, CH 8057 Zürich, Switzerland

**Keywords:** Locomotion, Gait disturbance, Cattle, Bovine, Cow welfare

## Abstract

**Background:**

Lameness in dairy cows has been an ongoing concern of great relevance to animal welfare and productivity in modern dairy production. Many studies have examined associations between various factors related to housing, management, and the individual animal and the occurrence of lameness. The objective of this systematic review was to answer the research question “what are risk factors associated with lameness in dairy cows that are housed in free stall barns or tie stall facilities”. Furthermore, we performed a synthesis of current evidence on certain risk factors by means of a meta-analysis to illustrate the strength of their association with bovine lameness.

**Results:**

Following pre-defined procedures and inclusion criteria in accordance with the PRISMA statement, two observers independently included 53 articles out of a pool of 1941 articles which had been retrieved by a broad literature research in a first step. 128 factors that have been associated with lameness were identified in those papers. Meta-analyses were conducted for five factors presented in six different studies: Body condition score, presence of claw overgrowth, days in milk, herd size, and parity. Results indicated that a body condition score of ≤2.5/5 is associated with increased odds of lameness. A higher risk of being lame was found for the presence of claw overgrowth, the first 120 days in milk, larger herd sizes, and increasing parity. Throughout the study, we encountered profound difficulties in retrieving data and information of sufficient quality from primary articles as well as in recovering comparable studies.

**Conclusions:**

We learned that an abundance of literature on bovine lameness exists. To adequately address a problem of this importance to both animal welfare and economic viability, solid evidence is required in the future to develop effective intervention strategies. Therefore, a consistent working definition of lameness and specific risk factors should be an option to consider.

## Background

Ranking third after reproductive failure and mastitis, bovine lameness is one of the principal economic and animal welfare issues in modern intensive dairy production all over the world [1–3]. Stanek [4] has described the condition as an inability to express a normal and functional gait pattern in one or more limbs usually as a consequence of pain. Multiple approaches have been established over the years to identify lame animals based on different characteristics of locomotion [5–7]. Research has indicated that lameness in dairy cows has a pronounced adverse effect on milk production [8–10], reproductive performance [11–13], longevity [14], and general well-being [15]. Furthermore, it is a painful condition [16, 17] that impairs the natural behavior of affected animals [18, 19]. The European Food Safety Authority (EFSA) has presented an insightful report of factors associated with lameness in dairy cows emphasizing that the housing environment of cattle is of crucial importance in the context of lameness development [20, 21]. Concomitantly, Bell et al. [22] have introduced a control program based on the principles of hazard analysis and critical control points (HACCP) to tackle lameness in dairy heifers. However, subsequent investigations on prevalence in North America and in Europe have clearly corroborated that lameness still is an ongoing concern [23].

Reviews have been published on lameness in dairy cows, approaches to detect lame animals, the treatment and prevention of lameness and digital dermatitis [24, 25] and the role of the environment on lameness dynamics [26–28]. The number of systematic reviews is yet still short and to our knowledge, neither a systematic review nor a meta-analysis has so far been conducted to evaluate risk factors associated with lameness in dairy cows. Against this background, the objective of the present work was to address the research question “what are risk factors associated with lameness in dairy cows that are housed in free stall barns or tie stall facilities” and to give a careful compilation and a statistical evaluation of literature by means of a systematic review and meta-analyses. We aimed to contribute evidence to the current knowledge by giving an intricate overview of literature as well as by providing a summary estimate of risk factor effects. Furthermore, areas of lack of knowledge were to be identified and outlined.

## Results

### Systematic review

Additional files 9 and 10 contain the data sets used for the systematic review and for the meta-analyses. Additional file 11 includes the references to all studies listed in Additional files 2, 3, and 4.

**Fig. 1:**
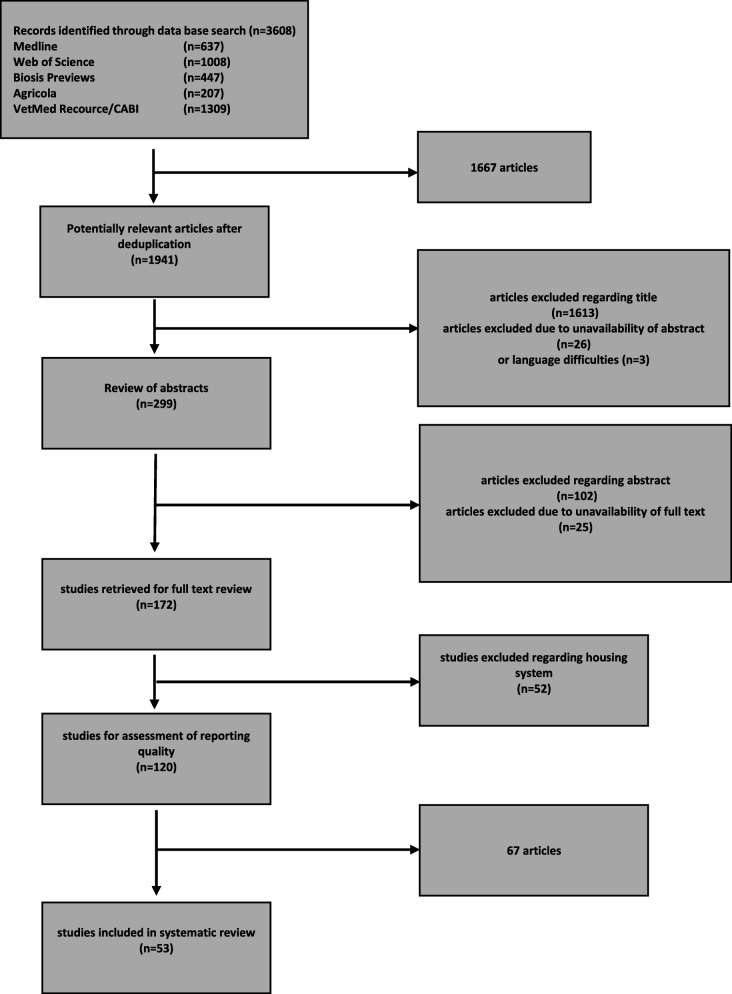
PRISMA flow chart of literature research and study selection process at different stages of the review process

A PRISMA flow chart was generated in order to present an overview of literature search and study selection at various stages of the review process (Fig. 1). Literature research of five electronic sources yielded a pool of 3608 references altogether of which 1941 remained within the analysis after deduplication (Table 1). A total number of 1613 publications were excluded on the basis of their title, the abstract of 26 articles was not available and three publications had to be excluded due to language difficulties (Japanese, Polish, Turkish). Subsequently, abstracts of 299 remaining articles could be examined, whereby 25 were not accessible by any means and 102 were excluded. Full texts of 172 publications were hence thoroughly reviewed. At this stage, 52 studies exited the subsequent review process as no cows were housed in either tie stall facilities or free stall barns. Information on the study design and housing conditions of these 52 studies excluded due to housing are provided in Additional file 1.

**Table 1:** Number of extracted papers per data base

Data Base	Deduplication	
	Before	After
MEDLINE (incl. Epub ahead of print, In process & other non-indexed citations	637	192
Web of Science	1008	990
Biosis Previews	447	45
AGRICOLA	207	11
VETMED RESOURCE/CABI (https://www.cabi.org/VetMedResource/)	1309	703
Pool	3608	1941

For each data base, the number of articles is shown before and after deduplication. 3608 articles were retrieved in the first place, of which 1941 were retained for further analysis after deduplication. In the VETMED RESOURCE/CABI database, 1531 articles were found, but only 1309 of these could be extracted

120 articles were assessed for reporting quality using the STROBE checklists [29]. Additional file 2 provides insights into the application of the STROBE guidelines for each of the 120 scrutinized publications. Also, information on whether an article was included in the review or entered the stage of meta-analyses is provided. During this las step, 67 studies were excluded. The most common problems of reporting quality identified from the STROBE checklist in these publications were the elaboration on the study design, i.e. the type of study (Item No. 1a), as well as reporting of eligibility criteria (Item No. 6), potential sources of bias (Item No. 9), and the elaboration on limitations or critical overall interpretation of the results (Item No. 19, Item No. 20, Item No. 21). Also, in a considerable number of publications, information on sample size and number of subjects at each stage of the study (Item No 6. and especially Item No. 13) were missing. For a more detailed overview, we refer to Additional file 2.

We included 53 studies in the systematic review (Additional file 3). Within these, 128 risk factors associated with lameness in dairy cows were identified (Additional file 4). Additional file 4 also provides information on the number of studies per risk factor.

Considerable heterogeneity was present in the definition of lameness and the assessment of dairy cow locomotion among studies. Lameness has been described as the inability to express a physiological locomotion pattern in one or more limbs most frequently as a consequence of pain [4, 30–32]. Further definitions of lameness have been introduced by individual research as well. Moreover, in some cases, lameness has been regarded as an equivalence of the presence of certain claw-associated conditions or the fulfilment of a certain score [33–35]. Also, some studies have not outlined a specific definition of lameness [36]. This considerable heterogeneity of nomenclature was also present in the definition of lameness among studies examined in the course of this work.

Based on the literature screened, 18 different approaches could be identified in the present study. These can be seen in Additional file 3 (4th column of the table). Whereas most studies adhered to the introduced scoring systems and the criteria for classifying a cow as lame, some studies integrated additional criteria or modified existing locomotion scoring systems.

### Meta-analyses

Table 2 gives an overview of risk factors and studies that were included in meta-analyses.

**Table 2:** Risk factors and studies included in meta-analyses

Risk factor	Author(s)	Year	Study
BCS	King et al.	2017	Cow-level associations of lameness, behavior, and milk yield of cows milked in automated systems
	Solano et al.	2015	Prevalence of lameness, claw lesions, and associated risk factors in dairy farms in Selangor, Malaysia
Claw overgrowth	Sadiq et al.	2017	Prevalence of lameness, claw lesions, and associated risk factors in dairy farms in Selangor, Malaysia
	Solano et al.	2015	Prevalence of lameness and associated risk factors in Canadian Holstein-Friesian cows housed in freestall barns
DIM	Sadiq et al.	2017	Prevalence of lameness, claw lesions, and associated risk factors in dairy farms in Selangor, Malaysia
	Manske T.	2002	Hoof lesions and lameness in Swedish dairy cattle: prevalence, risk factors, effects of claw trimming, and consequences for productivity
Herd size	Yaylak et al.	2010	The effects of several cow and herd level factors on lameness in Holstein cows reared in Izmir Province of Turkey
	Alban, L.	1995	Prevalence of lameness and associated risk factors in Canadian Holstein-Friesian cows housed in freestall barns
Parity	King et al.	2017	Cow-level associations of lameness, behavior, and milk yield of cows milked in automated systems
	Sadiq et al.	2017	Prevalence of lameness, claw lesions, and associated risk factors in dairy farms in Selangor, Malaysia
	Solano et al.	2015	Prevalence of lameness and associated risk factors in Canadian Holstein-Friesian cows housed in freestall barns
	Yaylak et al.	2010	The effects of several cow and herd level factors on lameness in Holstein cows reared in Izmir Province of Turkey
	Manske T.	2002	Hoof lesions and lameness in Swedish dairy cattle: prevalence, risk factors, effects of claw trimming, and consequences for productivity
	Alban, L.	1995	Prevalence of lameness and associated risk factors in Canadian Holstein-Friesian cows housed in freestall barns

A Body Condition Score (BCS of ≤2.5 was regarded as reference category and.

**Fig. 2:**
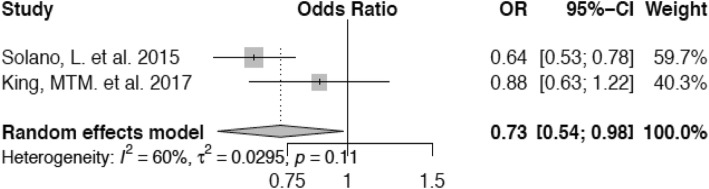
Forest plot for BCS 3.0 vs. BCS ≤ 2.5. Cows with a BCS of 3.0 are at decreased odds of lameness (OR 0.73) compared with animals in the reference category

**Fig. 3:**
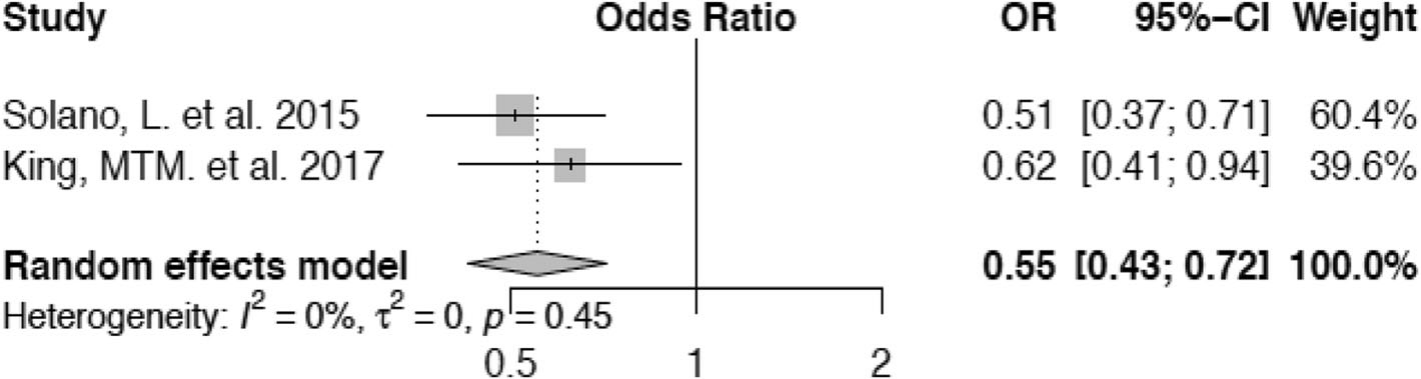
Forest plot for BCS ≥ 3.5 vs. BCS ≤ 2.5. Animals with a BCS of ≥3.5 have lower odds to become lame (OR 0.55) than cows with a BCS of ≤2.5

For the meta-analysis of the association of body condition with lameness, we were able to include two studies. As for Solano et al. (23), the only BCS category presented in the article, that was comparable to a BCS of 3.0 in King et al. [37] was 2.75–3.25, which we regarded as equivalent. Additionally, information on the number of lame and sound animals in each BCS group was extracted from a bar plot diagram as precisely as possible. Furthermore, we determined a BCS of ≤2.5 as the reference category for both studies and calculated the values for King et al. [37] to render both studies combinable. Scores of 3.0 and ≥ 3.5 were compared with this. Cows with a BCS of 3.0 show a decreased risk (Odds Ratio (OR) 0.73; confidence interval (CI) 0.54–0.98) to develop lameness compared with those animals in the reference category (Fig. 2) and cattle with a condition score of ≥3.5 are at lowest risk of lameness (OR 0.55; confidence interval 0.43–0.72) in comparison with those within the group of cows with a BCS of ≤2.5 (Fig. 3).

**Fig. 4:**
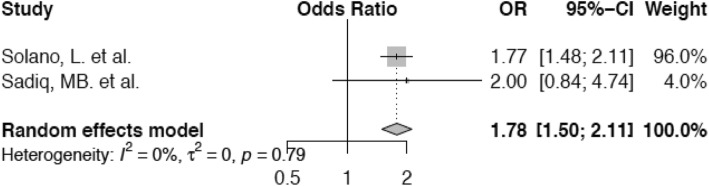
Forest plot for presence of claw overgrowth vs. absence of claw overgrowth. The presence of claw overgrowth increases the odds of being lame (OR 1.78) in an individual animal

The absence of claw overgrowth was the reference category and we examined the risk for lameness in cows with overgrown claws (Fig. 4). Cows with overgrown claws have increased odds (OR 1.78; confidence interval 1.50–2.11) of lameness compared with animals the claw of which are of normal shape.

**Fig. 5:**
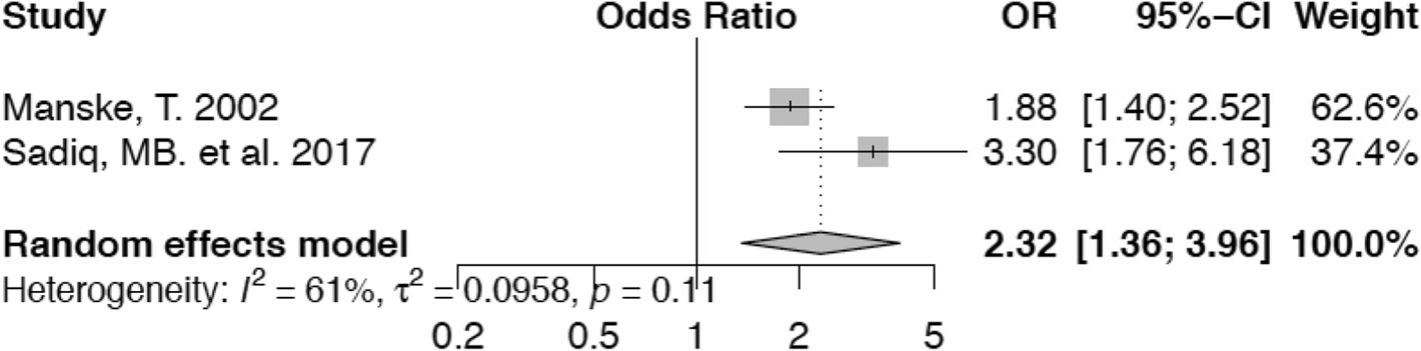
Forest plot for cows 120 DIM vs. animals > 120 DIM. The first 120 days of lactation represent a risk period for the occurrence of lameness, increasing the odds by a factor of 2.32 compared with animals in the reference category

Figure 5 shows that the risk of lameness is higher (OR 2.32; confidence interval 1.36–3.96) for cows during the first 120 days of milk than for animals in a later stage of lactation.

**Fig. 6:**
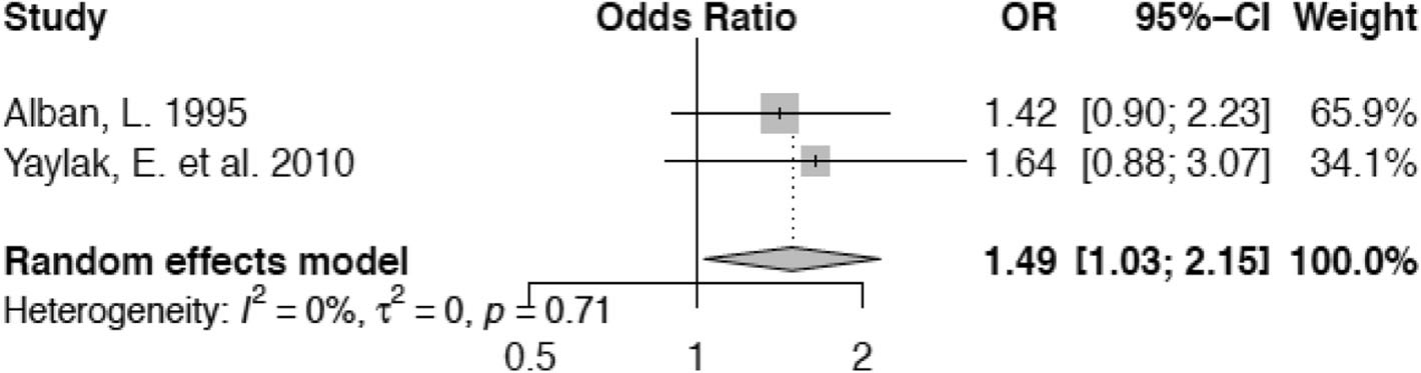
Forest plot for herd size of 30–50 animals vs. ≤ 29 animals. Animals kept in herds of 30–50 cows have a higher risk for lameness (OR 1.49) than cows in smaller herds (≤ 29 cows)

**Fig. 7:**
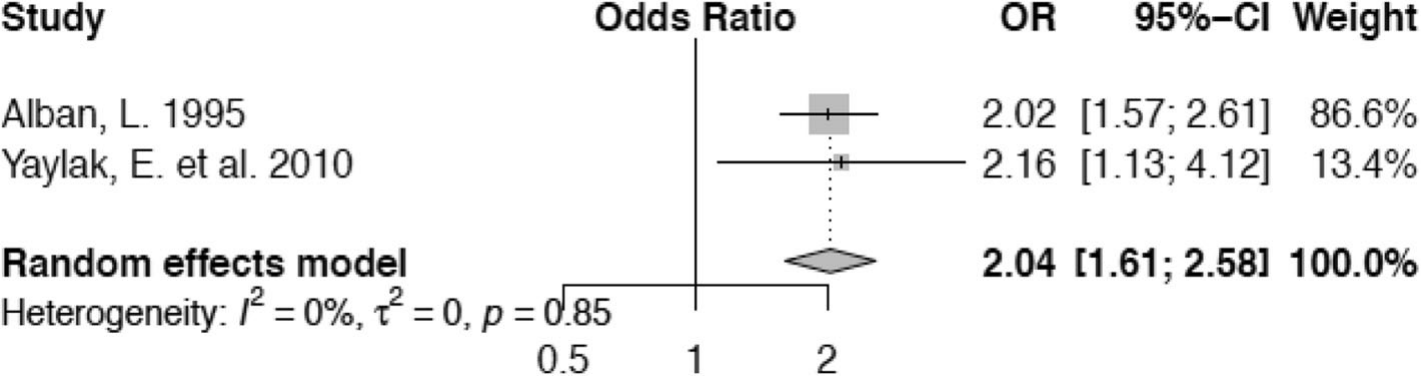
Forest plot for herd size of ≥50 animals vs. ≤ 29 animals. A herd size of ≥50 cows increases the odds of lameness by a factor of 2.04 compared to cows living in herds of ≤29 animals

Lactating cow herd sizes of 30–50 animals or 50 or more animals, respectively, increase the odds of becoming lame (OR 1.49; confidence interval 1.03–2.15 and OR 2.04; confidence interval 1.61–2.58) compared with herd sizes of ≤29 animals (Figs. 6 and 7).

**Fig. 8:**
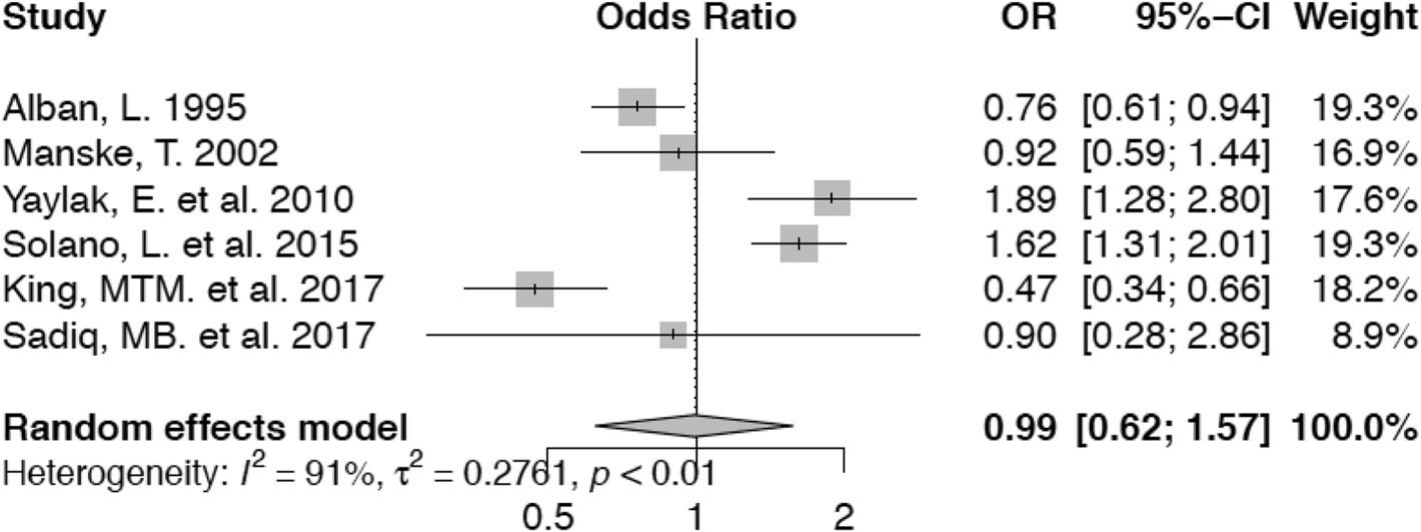
Forest plot for parity 2 vs. parity 1. Parity 2 protects cows from being lame compared with animals in parity 1 (OR = 0.99)

**Fig. 9:**
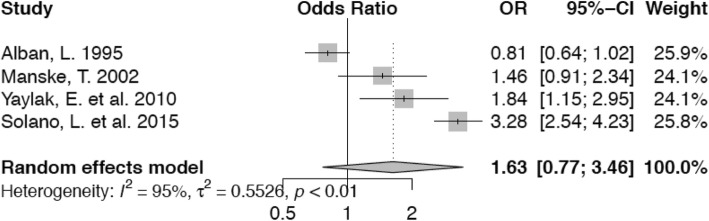
Forest plot for parity 3 vs. parity 1. Cows in parity 3 have higher odds (OR 1.63) of lameness than animals in the reference category

**Fig. 10:**
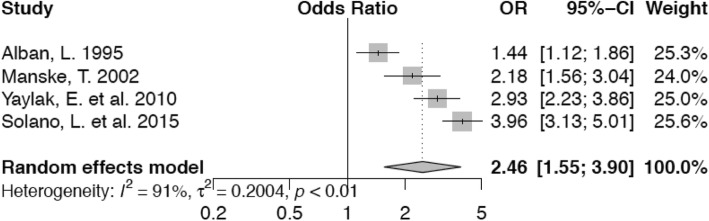
Forest plot for parity 4+ vs. parity 1. Cows in parity 4 or higher are at an augmented risk for lameness (OR 2.46) compared with animals in parity 1

Animals in the second lactation have nearly the same odds (OR 0.99; confidence interval 0.62–1.57) of lameness in comparison with those in parity 1 (Fig. 8). This is not statistically significant. Cows in their third parity on the other hand, have an non-significantly increased risk (OR 1.63; confidence interval 0.77–3.46) for lameness (Fig. 9) and the risk of lameness for those animals in fourth or higher parity is significantly higher (OR 2.46, confidence interval 1.55–3.90) compared with animals in their first lactation (Fig. 10).

### Assessment of bias

The ROBINS-E tool (version 2017) [38] was implemented to assess bias in the six primary articles that entered the stage of meta-analyses. The results of this assessment are displayed in Additional file 5 for each of the six studies.

Furthermore, funnel plots for the assessment of publication bias can be seen in Additional file 6. The graphs appeared to be mainly symmetrical and evenly distributed, although dots were not entirely located within the funnel itself. In the case of slight asymmetry, studies with larger sample sizes appeared to report outcomes closer to no effect. It is recommended to be cautious with the interpretation of the funnel plots due to the small number of incorporated studies.

## Discussion

### Systematic review: findings

The objective of this study was to give a comprehensive overview of risk factors for lameness in dairy cows on the one hand and to quantitatively synthesize information of the existing body of research on the other hand. Furthermore, we intended to present potential areas where knowledge should be increased and to perform meta-analyses if possible. Out of 1941 studies retrieved initially, we were able to identify 128 risk factors that have been associated with lameness in dairy cows in a total number of 53 articles. For five different risk factors in six of these papers, we were able to retrieve sufficient information to perform meta-analyses to elicit the strength of their association with lameness.

Well-defined locomotion scoring systems have been developed to record characteristics and aberrations of dairy cow gait and to quantify lameness problems in a herd. 18 different approaches to assess locomotion in dairy cows could be identified in the present work. It may be problematic to appraise and compare the outcomes of different research projects if definitions and approaches vary distinctly across studies. For future investigations, following a precise and consistent nomenclature when addressing the same problem is therefore recommended. This may be achieved by an international group of epidemiologists and experts in the field similar to those efforts undertaken in order to establish tool for the assessment of bias in primary studies [39–41]. For example, for mastitis in dairy cattle, definitions and guidelines for diagnosis have been established as early as 1987 [42].

The large number of articles (1941) extracted from the data bases provides compelling evidence that lameness in dairy cattle has been an ongoing concern of substantial importance. Even though literature has introduced an extensive number of studies related to the issue, we were only able to include 53 of the original 1941 studies in the systematic review and six in meta-analyses.

Firstly, this is due to the fact that studies where cows were housed in facilities other than tie stalls or free stalls were excluded from the current analysis in order to increase comparability. Secondly, a large variety of studies has described risk factors which is also emphasized by the multifactorial nature of dairy cow lameness. Thirdly, we had to exclude a total of 51 studies, because either abstracts or full texts were not accessible and three more publications had to be excluded due to unavailability in any of the languages we understand. For modern research, it is absolutely indispensable that knowledge is broadly accessible in order to be shared, understood and taken into consideration by a wide audience. New insights into existing problems can hence be implemented and built on properly and quickly.

Regarding the meta-analyses, studies that did not provided the data required for the approach chosen for this work were excluded. Accordingly, only 6 out of 53 articles included in the systematic review, entered the stage of meta-analyses.

Regarding certain publications, we had to calculate standard errors from confidence intervals, if only the latter were reported. This resulted in an approximation of the actual values only and may be a weakness of this study. We need to underscore that we chose this approach due to incomplete documentation and presentation of primary data in the articles we had retrieved. We are however convinced that the eventual outcome has not been negatively influenced by this approach In general, it may be conducive from an evidence-based point of view if data were uploaded as soon as a study is published.

### Systematic review: methodology and limitations

Even though systematic reviews reflect the best evidence, the approach is susceptible to the introduction of bias owing to the retrospective character of the analysis and the study selection process [43, 44]. Bias can enter in various forms and at all stages throughout the review process [45–47]. Therefore, minimization and prevention of bias was attempted on several levels in the present study. Three previously designed quality assessment tools, i.e. AMSTAR, PRISMA, and ROBIS, for the appraisal of systematic reviews have been oriented towards throughout the conduct of this study [46, 48, 49].

The ROBIS tool for the assessment of bias in systematic reviews has placed clear emphasis upon the importance to determine areas where bias may have entered the systematic review [46]. It is therefore important to note that certain flaws might be present in the methodology of this review. The PRISMA guidelines [49] demand an open a priori registration of systematic reviews. Unfortunately, at the inception of this study, the authors did not find a possibility to register a pre-specified protocol to this systematic review. Therefore, the protocol is attached in Additional file 7. Other veterinary research groups have overcome this limitation by publishing the study design prior to completing this study [39, 50].

Moreover, the exact study type had not been determined in preparation of the present study. It had furthermore been expected that controlled field trials may not be abundant in the research area this work focused on. Throughout the course of this study, observational studies appeared to be the predominant type of study in the context of dairy cow lameness and associated risk factors which corroborated our initial expectation. This is in alignment with findings by other systematic reviews that were equally unable to include larger number of studies in the summary and synthesis due to a lack of interventional studies and field trials [24, 25]. However, in order to ensure a systematic review process, a previously specified and clearly defined agenda was followed which involved distinct information on the research topic and population of interest, i.e. risk factors associated with lameness in dairy cows housed either in free stall barns or tie stall facilities.

Also, even though full-text screening of studies, data extraction, and the implementation of the STROBE checklists to assess the reporting quality of primary articles were performed by one single reviewer, these procedures were discussed before starting the review process. Moreover, the second reviewer checked the decision upon inclusion after full texts had been screened in alignment with the procedures presented by Whiting et al. (2016) [46] and an a priori discussion including a biostatistician and an epidemiologist was held in order to determine which data are to be extracted from the included studies. Finally, since the STROBE checklists provide an objectified and clearly comprehensible tool to appraise studies, the fact that one reviewer independently assessed primary articles may only be a minor source of potential bias. The choice that 15 criteria were necessary for further inclusion was subjective decision to the extent that 15 out of the 22 criteria appeared to be a critical number of criteria that could be met within the primary articles. This subjective, yet not arbitrary choice may be regarded as a source of bias in the present review. Given the fact that distinct definitions or consistencies are largely absent in the context of lameness in dairy cows, it may appear reasonable to accept subjectively made compromises where necessary.

### Bias

The assessment of bias in observational studies is rather challenging [51]. However, addressing potential bias in primary articles of observational studies is important and ought to be paid attention to when performing a systematic review. In recent years, international efforts have been undertaken in order to develop tools for the assessment of bias in primary articles [38, 52, 53]. The ROBINS-E tool (version July 2017) has been presented for the assessment of ‘Risk of Bias in Non-randomized Studies of Exposures’. This tool focuses on seven domains of potential bias within a publication: confounding, selection of participants into the study, classification of exposures, departures from intended exposures, missing data, measurement of outcomes, and selection of the reported results. In the context of these domains, ‘signalling questions’ are provided addressing several aspects of bias [38, 51, 54]. Within the seven domains, conclusive judgements allow for the summary of bias assessment. In the present study, we implemented ROBINS-E in the form of the preliminary risk of bias for exposures tool template [38] in order to asses bias in the six studies that entered the meta-analyses. To our knowledge, this is the first systematic review in the veterinary literature to use ROBINS-E in this context. Since this is a very novel approach to address the identification of bias within a systematic review, the ROBINS-E tool has been under development an needs further refinement in order to provide a practical basis of evaluation [51]. This is the reason why we also encountered some difficulties in the application of the tool to the six studies included in our meta-analyses. However, ROBINS-E may be of invaluable help to the work of future systematic reviews and ought to be taken into consideration.

Funnel plots were created for each single meta-analysis in order to assess the presence of potential publication bias across the primary investigations. The assessment of publication bias is yet limited by the fact that many meta-analyses only incorporate a small amount of primary investigations and the symmetry of funnel plots may be treacherous as soon as less than 10 studies are combined in a meta-analysis [55, 56]. Hence, it is important to be cautious since the number of studies included within the analyses of the current work is small with a maximum of six studies in only one of the present meta-analyses. For this reason, funnel plots were evaluated visually not statistically. Due to this insufficient statistical power, publication bias cannot be excluded.

### Meta-analyses

Meta-analyses were performed for six references of the final systematic review. This short number of studies that entered the stage of meta-analyses is due to the fact, that in the major part of articles, the data required for the meta-analysis approach chosen for this study were not available. The short number can also be traced back to the fact that either calculation of the required parameters from information within the primary articles was not possible or that in one out of the five cases the corresponding authors contacted to retrieve date were able to provide this information.

In the present study, the random effects model was chosen to display outcomes for each individual meta-analysis. Random effects meta-analyses provide the average effect across all studies within the approach and acknowledge that effects may differ across studies and possibly unexplained heterogeneity may be present [57]. The percentage of heterogeneity within a meta-analysis, i.e. the value of I^2^ hence gives an indication of the variability in effect estimates as a consequence of true differences between studies rather than chance [57, 58]. This can be attributed to differences in study settings, populations, and other factors or chance in the course of sampling. By contrary, fixed effects meta analyses assume that all studies share one common effect size and no heterogeneity is present between studies [57–59]. Potential variation are hence solely a consequence of chance during the sampling process. Fixed effect models are adequate for the synthesis of a small number of well-controlled, functionally similar studies with identical settings [58, 59]. Generalizations to populations are not intended but rather to address a specific population. Since random effects meta-analyses acknowledge the presence of heterogeneity and because they have been the most common approach in a medical context [57], this approach was chosen. In order to correctly interpret the results, it is important to consider that on an individual study basis, the effect of a certain risk factor may be different from the average effect estimate yielded in the random effects meta-analysis.

The choice of the reference category in separate risk factor studies was fairly unequal among studies and since it is necessary for meta-analyses to determine the reference category to be able to combine evidence from various studies, we had to apply the Mantel-Haenszel method to pool odds ratios. This may be understood as a weak point of our study but is due to variable categorization in the included studies.

Non-infectious pathologies of the claw particularly appear to be initiated by low body condition [40]. It has been discovered that thickness of the digital cushion is profoundly linked to body condition and decreases correspondingly to a decline in body condition [41]. Deeper structures, e.g. the corium, of the claw are hence less shielded from forces and pressure of weight-bearing [41, 60] and become more susceptible to damage and lameness-causing conditions such as sole ulcers and white line disease as a consequence of disruption of claw horn growth. Randall et al. [61] have therefore suggested to keep cows at a BCS of at least 2.5 for the best results in reducing lameness. This is in compliance with the results of the present meta-analysis for BCS and its association with lameness. When interpreting our results we recommend to acknowledge that the procedures of data extraction may represent potential limitations to this particular meta-analysis. Apart from that, an additional element regarding the association between low body condition and lameness may be decreased feed intake in lame cows as they are either less able to compete with sound herd mates or modify their behavior and spend a larger amount of time lying down [17, 62, 63]. The association between BCS and lameness is likely to be part of a vicious circle and mutual causality seems rather reasonable in this context.

Claw overgrowth is positively associated with lameness in dairy cattle [23, 64] and claw trimming management hence constitutes a crucial point in managing foot health in dairy cows. It is important to consider that claw overgrowth was assessed subjectively without the implementation of an established or validated scoring system in the primary studies included in this meta-analysis. This may have been a potential source of bias. The results of our meta-analysis further corroborate the evidence that claw overgrowth increases a cow’s risk of lameness. Not only are biomechanics positively influenced by claw trimming as weight load is more evenly distributed, but hoof growth characteristics are equally improved as horn growth is enhanced and wear attenuated [65, 66]. Lameness problems within a herd can therefore be effectively addressed by implementing correct functional claw trimming in adequately regular intervals [3, 67].

Our meta-analysis indicates that cows during the first 120 DIM have a higher risk of lameness (OR 2.32) than animals after that period. The initial four months after parturition challenge a cow’s ability to adapt to husbandry changes and associated environmental and nutritional conditions [68]. These factors in combination with the transition from late pregnancy to the onset of lactation may facilitate the development, emergence and deterioration of claw lesions. High milk yield at the onset of lactation may be an important additional factor to exacerbate the situation by promoting increased loss of body mass after parturition [41]. Digital cushion thickness decreases correspondingly and renders animals more susceptible to claw diseases, which may result in lameness. Reduction in feed consumption secondary to lameness may further aggravate the problem.

Equivocal results have been presented in regard to the association between lameness and herd size. According to several studies, a lower prevalence of lameness in larger herds reflects more professional lameness management procedures [23, 69, 70], i.e. automated production elements and additional staff for lameness detection and treatment. Similar observations have been reported by Adams et al. [71]. Richert et al. [72] have yet not recognized a positive association between larger herd size and lameness prevalence. Alban [35] hypothesized that producers may spend less time observing their animals in larger herds as a consequence of mechanization of process steps. In larger herds, usually fewer qualified personnel per cow are available [73] and individual animals may hence be watched less intensively.

Our meta-analysis on the association between herd size and lameness supports the view that larger herd size increases the odds of lameness for an individual animal. The reasons may be as outlined previously, but we need to emphasize, that our analysis was based on 2 European studies with a rather small overall herd size even in the group of large herds compared with other studies particularly from North America [23, 71]. Different causalities in conjunction with differing operational structures on a farm may be present on large-scale farms in North America. For herd size, we therefore recommend to evaluate studies from Europe and North America independently. Furthermore, the two studies included in our meta-analysis were conducted some decades apart from each other which underscores the necessity to be cautious when interpreting the results. Additionally, when assessing the impact of herd size on the risk of lameness, overstocking has to be taken into consideration as an important factor as well. This may be the true underlying problem, since the absolute number of animals within a herd reflects a different situation than the number of cows in relation to the number of free stalls or available feeding space, respectively.

Higher parity increases a cow’s risk of being lame [74–76]. Multiparous cows have obviously been confronted with the confined artificial environment they are housed in for a longer time and a cumulative effect of calving associated stress, metabolic changes throughout parities and housing-related deficiencies may be detrimental to hoof conformation and claw health and add up to existing problems. Milk yield may also play an important role in this context considering that production levels usually increase as lactation number progresses [77].

This is basically consistent with the results of our meta-analysis of the impact of parity on the risk of lameness for cows in parity 4+. Cows in parities 4 and higher have 2.46 times increased odds of being diagnosed as lame, respectively compared with first lactation animals. As for parities 2 and 3, we infer that cows basically do not differ from first-lactation animals due to the fact that the result of the meta-analysis is not significant.

An abundance of factors influence lameness in dairy cattle and yet additional light has to be shed on many interrelationships and mechanisms. Out of 128 risk factors, we were able to collect data and produce evidence on the impact of five different risk factors on lameness in dairy cows. In the course of this study, it has become increasingly apparent that despite the extensive body of research on bovine lameness and associated risk factors, only a few studies remain comparable. The interpretation of individual study outcomes may thus be challenging. Bovine lameness as a multifactorial disorder still is a major issue in dairy production that requires additional research in the future, preferably in a standardized way.

## Conclusions

Lameness is a tremendous problem of the modern dairy industry. Solid evidence is needed to further tackle this issue properly, in order to improve and ensure animal welfare, longevity and economic viability. The results of our work clearly show that we encountered difficulties in collecting and extracting data completely, because articles did not provide sufficient information and we had to apply elaborate strategies in order to receive a comprehensive selection of data we were able to work with. Regardless of these challenges, the present study provides a collation of risk factors of lameness in dairy cows on the one hand and evidence on the strength of the association of five different factors with lameness on the other hand. Our analysis is supposed to aid future studies on where to place emphasis on regarding study design. A joint initiative consisting of experts in the field and epidemiologists may be an option to establish consistent working definitions and well-founded study design, analysis, and reporting. This could help improving dairy cow welfare, facilitate maintaining economic efficiency, and reduce the generation of “research waste”.

## Methods

This systematic review and meta-analyses were conducted following a pre-specified study protocol in compliance with the procedures presented by Shamseer et al. [78] (Additional file 7). Furthermore, three commonly implemented quality assessment tools for systematic reviews and meta-analyses, i.e. AMSTAR, PRISMA, and ROBIS, were taken into consideration throughout the course of this study [46, 48, 49].

### Search strategy and selection criteria

A professional librarian experienced with electronic sources conducted an extensive literature research for all available years from inception up to February 27, 2018, using the search engines MEDLINE (incl. Epub ahead of print, In process and other non-indexed citations), Web of Science, BIOSIS Previews, AGRICOLA, VETMED RESOURCE/CABI.

The search terms listed below were applied to extract as many potentially relevant articles as possible from the electronic sources. The search terms were separated into 4 components in correspondence with the elements of this review: risk factors, lameness, dairy cows. Alternative wording was permitted for each of these components, indicated by the operator “OR” and every component was combined with the others by the separator “AND”. An asterisk indicates that the data base will be scrutinized for words beginning with these letters.

1. To identify studies with a study population of animals in the dairy sector exclusively. Alternatively to “dairy cow” other wording was permitted by the operator “OR”.

- (“dairy cow” OR “dairy cows” OR “dairy farm” OR “dairy farms” OR “dairy herd” OR “dairy herds” OR “dairy cattle”) **AND:**

2) To identify studies with the relevant outcome of lameness. Alternative wording was permitted by the operator “OR”.

- (lame* OR ((impaired OR alter* OR disturb*) **AND:**

3) To identify all possibly relevant studies describing locomotion characteristics.

- (gait OR locomotion))) **AND:**

4) To identify studies describing various factors associated with lameness. Alternative wording was permitted by the operator “OR”.

- (((risk OR management OR “herd-level”) **AND** factor*) OR prevalence OR associat*)

### Study selection

Initially, studies of all designs and of all languages describing risk factors for lameness in dairy cows and alternative wording were admitted according to the search terms outlined above. Subsequently, studies which were not written or available in Dutch, English, French, German, Italian, Portuguese or Spanish were excluded from further assessment as well as publications that were not accessible by any means. Full texts were subjected to screening and we included only those studies where animals were kept in free stall facilities or tie stall operations. If a publication compared lameness in two different housing systems where one of the systems was either a tie-stall or free-stall, this publication was not directly excluded but entered the stage of the assessment of reporting quality. Also, studies were admitted to the next stage if groups of cows where housed in either a tie-stall or a free stall barn and other groups of cows were housed in a different housing system.

After exclusion of duplicate studies, two reviewers (AOE, AS) independently examined titles and abstracts of all remaining publications in compliance with the eligibility criteria. When disagreement about the eligibility of an article arose, a third investigator (GKS) was consulted to decide upon inclusion. Where a study appeared to be eligible, the full text was obtained and examined for eligibility one more time.

The primary investigator (AOE) assessed the reporting quality of each study using the STROBE checklists [29]. Studies that did not comply with at least 15 of the 22 listed criteria in these guidelines were excluded from subsequent scrutiny. Also, non-primary studies, review, conference abstracts or book sections were excluded as well.

### Data extraction

The primary reviewer (AOE) extracted data regarding author and year of publication, country, risk factors for lameness in dairy cows, definition of lameness and applied locomotion scoring system, number of animals, housing system and funding of the research project. Type of extracted information had been previously specified in consultation with a biostatistician (AR) and an epidemiologist (SH). When relevant data were missing, the corresponding author was contacted to access further information.

### Statistical analysis

Data were extracted and collected using a single electronic form containing information on risk factor, author(s), study title, year of publication, country, total number of animals, group sizes i.e. absolute number or percentage of lame and sound animals with regard to different risk factors, confidence intervals, standard errors of odds ratios and coefficients, odds ratios and *p*-values using Microsoft Excel 2016 (macOS) [79].

All meta-analyses were carried out with the assistance of a biostatistician (AR). The R-package “meta” was applied for the following variables: BCS, DIM, claw overgrowth, herd size, and parity [80, 81]. The random effects model was chosen due to the underlying heterogeneity in population characteristics. The R function “metagen” was used to generate pooled estimates which were visualized in forest plots. Forest plots incorporated information on the OR and the 95% confidence interval of the summary effects. The shaded box represents the relative contribution of each study to the summary OR. Publication bias was assessed by creating funnel plots for each single meta-analysis using the R function “funnel” (see Additional file 6).

The meta-analysis approach implemented in this study required information on log(OR), standard errors of coefficients, and the number of lame and sound animals in each category of the risk factor in all meta-analyses. A meta-analysis was performed if sufficient and usable data on a risk factor could be retrieved from a primary article. For five studies, corresponding authors were contacted in case the information was not available in the published paper.

Coefficients (log(OR)) were extracted directly from the articles or obtained by means of transforming the reported odds ratios with natural logarithm. If information on standard error was not available in a particular paper, we calculated the value from confidence interval limits if reported. Confidence intervals around the coefficients were used directly for 95 and 90% confidence intervals according to Higgins et al. [82].

For BCS, we had to change the reference category to a reference category different from the original category in King et al. [37]. The scoring system suggested by Edmonson et al. [83] has been widely used across studies. We determined a BCS of ≤2.5 as the reference category according to the majority of studies about BCS and lameness and calculated odds ratios and standard errors for the other categories of BCS 3.0 and BCS ≥ 3.5, respectively, compared with a BCS of ≤2.5. The standard error was calculated using the formula in Additional file 8. If odds ratios had to be pooled, we implemented the Mantel-Haenszel method [58].

## Supplementary information

**Additional file 1.** Study design and housing conditions of 52 studies excluded at the stage of housing assessment.

**Additional file 2.** Application of the STROBE guidelines for each of the 120 scrutinized publications.

**Additional file 3.** Overview of the 53 studies included in the systematic review.

**Additional file 4.** 128 risk factors associated with lameness.

**Additional file 5.** Implementation of ROBINS-E for the assessment of bias in primary studies .

**Additional file 6.** Funnel plots for the assessment of publication bias for each meta-analysis .

**Additional file 7.** Pre-specified study protocol in compliance with the procedures presented by Shamseer et al. [78].

**Additional file 8.** Calculation of standard errors for the meta-analysis of BCS.

**Additional file 9.** Data set used for the systematic review .

**Additional file 10.** Data set used for meta-analyses.

**Additional file 11.** Reference list of studies listed in Additional files 2, 3, and 4.

## Data Availability

The datasets used in this work are available as additional files to this paper.

## Abbreviations

AMSTAR: A measurement tool to assess the methodological quality of systematic reviews
BCS: Body condition score
CI: Confidence interval
DIM: Days in milk
EFSA: European Food Safety Authority
HACCP: Hazard analysis and critical control points
OR: Odds ratio
PRISMA: Preferred reporting items for systematic reviews and meta-analyses
ROBINS-E: Risk of bias in non-randomized studies of exposures
ROBIS: Risk of bias in systematic reviews
STROBE: Strengthening the reporting of observational studies in epidemiology
