# Supplementary material for: A systematic review and meta-analyses of risk factors associated with lameness in dairy cows
Source: BMC Vet Res. 2019 Oct 16;15:346. doi: 10.1186/s12917-019-2095-2 (PMC6796431; doi:10.1186/s12917-019-2095-2)
Supplement: Supplementary file 1 — Additional file 1. Study design and housing conditions of 52 studies excluded at the stage of housing assessment. [file 12917_2019_2095_MOESM1_ESM.pdf]

Study design	Housing system	Number of studies in this category
Cohort study	No information on housing system	1
Experiment	Deep litter barn with straw bedding and access to pasture	1
	No information on housing system	1
	No stall, fully roofed, open cowsheds with dried manure bedding material	1
Longitudinal field trial	No information on housing system	1
Observational study	Loose/free housing (without further specification)	3
	Loose housing, brick paved (without further specification)	1
	No information on housing system	14
	Pasture-based	18
	Pasture-based + loose housing in straw pens	1
	Pasture-fed	2
	Zero-grazed (without further specification)	2
Review	No information on housing system	1
	Pasture-fed	2
Randomised controlled trial	No information on housing system	1
	Pasture-based	2
