# Supplementary material for: A systematic review and meta-analyses of risk factors associated with lameness in dairy cows
Source: BMC Vet Res. 2019 Oct 16;15:346. doi: 10.1186/s12917-019-2095-2 (PMC6796431; doi:10.1186/s12917-019-2095-2)
Supplement: Supplementary file 3 — Additional file 3. Overview of the 53 studies included in the systematic review. [file 12917_2019_2095_MOESM3_ESM.pdf]

Author(s), Year	Study	Country	Definition of Lameness/ Locomotion Scoring System	Number of animals	Housing system
Adams et al. (2017)	Associations between housing and management practices and the prevalence of lameness, hock lesions, and thin cows on US dairy operations	USA	Score ≥ 2 on a 3-point scale (1 = sound, 2 = mildly/moderately lame, 3 =severely lame)	22,042	Free stall Tie stall
Alban (1995)	Lameness in Danish dairy cows-frequency and possible risk factors	Denmark	Contusion, foul in the foot, sole ulcer, foot rot, interdigital dermatitis, laminitis, swollen hock, arthritis, other lameness	9,762	Free stall Tie stall
Alban et al. (1996)	Lameness in tied Danish dairy cattle: The possible influence of housing systems, management, milk yield, and prior incidents of lameness	Denmark	Foul in the foot (interdigital necrobacillosis), hock lesions (tarsal cellulitis and its sequelae)	26,499	Tie stall
Andreasen and Forkman (2012)	The welfare of dairy cows is improved in relation to cleanliness and integument alterations on the hocks and lameness when sand is used as stall surface	Denmark	Score ≥ 1 on a 3-point scale (0=not lame, 1=lame, 2=severely lame)	2,573	Free stall
Battagin et al. (2013)	Genetic parameters for body condition score, locomotion, angularity, and production traits in Italian Holstein cattle	Italy	Not specified	44,218	Free stall Tie stall
Becker et al. (2014b)	Lameness and foot lesions in Swiss dairy cows: II. Risk factors	Switzerland	Score ≥ 2 Sprecher et al. (1997)	1,449	Free stall Tie stall
Bernardi et al. (2009)	The stall-design paradox: Neck rails increase lameness but improve udder and stall hygiene	Canada	Not specified, scoring system by Flower and Weary (2006)	32	Free stall
Boettcher et al. (1998)	Genetic analysis of clinical lameness in dairy cattle	Canada/USA	Score ≥ 2 Wells et al. (1993a) Wells et al. (1993b)	1,342	Free stall
Bouffard et al. (2017)	Effect of following recommendations for tiestall configuration on neck and leg lesions, lameness, cleanliness, and lying time in dairy cows	Canada	Exhibition of ≥ 2 of the behaviours Leach et al. (2009), adapted by Gibbons et al. (2014)	3,436	Tie stall
Chapinal et al. (2013)	Herd-level risk factors for lameness in freestall farms in the northeastern United States and California	Canada/USA	Numerical Rating Score ≥ 3 Flower and Weary (2006), Chapinal et al. (2009)	14,112	Free stall
Chapinal et al. (2014)	Risk factors for lameness and hock injuries in Holstein herds in China	China	Numerical Rating Score ≥ 3 Flower and Weary (2006) Chapinal et al. (2009)	Not available	Free stall
Cook (2003)	Prevalence of lameness among dairy cattle in Wisconsin as a function of housing type and stall surface	USA	Score ≥ 3 on a 4-point scale 1=no gait abnormality, 2=slight lameness, 3=moderate lameness, 4=severe lameness	3,621	Free stall Tie stall
Cook et al. (2016)	Management characteristics, lameness, and body injuries of dairy cattle housed in high-performance dairy herds in Wisconsin	USA	Score ≥ 3 Sprecher et al. (1997)	9,690	Free stall
Dembele et al. (2006)	Factors contributing to the incidence and prevalence of lameness on Czech dairy farms	Czech Republic	Any degree of limping on one or more legs	Not available	Free stall
Dippel et al. (2009a)	Risk factors for lameness in cubicle housed Austrian Simmental dairy cows	Austria/ Germany	Score ≥ 3 Winckler and Willen (2001)	832	Free stall
Dippel et al. (2009b)	Risk factors for lameness in freestall-housed dairy cows across two breeds, farming systems, and countries	Austria/ Germany	Score ≥ 3 Winckler and Willen (2001)	3,514	Free stall
Espejo et al. (2006)	Prevalence of lameness in high-producing Holstein cows housed in freestall barns in Minnesota	USA	Score ≥ 3 Sprecher et al. (1997)	5,626	Free stall

Espejo and Endres (2007)	Herd-level risk factors for lameness in high-producing Holstein cows housed in freestall barns	USA	Score ≥ 3<br>Sprecher et al. (1997)	5,626	Free stall
Faye and Lescourret (1989)	Environmental factors associated with lameness in dairy cattle	France	Not specified	1,712	Free stall<br>Tie stall
Foditsch et al. (2016)	Lameness Prevalence and Risk Factors in Large Dairy Farms in Upstate New York. Model Development for the Prediction of Claw Horn Disruption Lesions	USA	Visual locomotion score ≥ 2<br>Bicalho et al. (2007a)	7,687	Free stall
Frankena et al. (2009)	The effect of digital lesions and floor type on locomotion score in Dutch dairy cows	Netherlands	Score ≥ 3<br>Manson and Leaver (1988)	225	Free stall
Green et al. (2010)	Associations between lesion-specific lameness and the milk yield of 1,635 dairy cows from seven herds in the Xth region of Chile and implications for management of lame dairy cows worldwide	Chile	Foot lesions causing lameness, diagnosed and recorded by farmers	1,635	Free stall
Green et al. (2014)	Temporal associations between low body condition, lameness and milk yield in a UK dairy herd	UK	Identifiable impaired mobility	1,.137	Free stall
Groehn et al. (1992)	Risk factors associated with lameness in lactating dairy cattle in Michigan	USA	any abnormality in locomotion as<br>determined by producer or farm veterinarian or both	3,610	Free stall<br>Tie stall
Gudaj (2012)	Associations between the occurrence of lameness, number of orthopaedic blocks used by hoof trimmers and management risk factors in dairy cow herds	Hungary	Score ≥ 3<br>Sprecher et al. (1997)	11,422	Free stall
Hedges et al. (2001)	A Longitudinal Field Trial of the Effect of Biotin on Lameness in Dairy Cows	UK	Not specified	900	Free stall
Hettich et al. (2007)	Factors associated to lameness in 50 dairy herds in the X-th Region, Chile	Chile	Sprecher et al. (1997), modified by Tadich et al. (2005)	7,501	Free stall
Hirst et al. (2002)	A mixed-effects time-to-event analysis of the relationship between first-lactation lameness and subsequent lameness in dairy cows in the UK	UK	Not specified	611	Free stall
Hultgren (2007)	Alley-floor design, claw lesions and locomotion in Swedish loose-housed dairy cattle	Sweden	Score ≥ 1<br>Sprecher et al. (1997), modified	183	Free stall
King et al. (2017)	Cow-level associations of lameness, behavior, and milk yield of cows milked in automated systems	Canada	Score ≥ 3<br>Flower and Weary (2006)	1,218	Free stall
King et al. (2016)	Associations of herd-level housing, management, and lameness prevalence with productivity and cow behavior in herds with automated milking systems	Canada	Score ≥ 3<br>Flower and Weary (2006)	1,230	Free stall
Manske (2002)[1]	Hoof lesions and lameness in Swedish dairy cattle: prevalence, risk factors, effects of claw trimming, and consequences for productivity	Sweden	Partly specified<br>Score ≥1 on a 3-point scale (0=sound, 1=mild variation from normal gait, 2=clearly favouring one or more limbs, 3=extremely unwilling to put weight on one or<br>more limbs or recumbent	4,899/<br>3,444/<br>2368[1]	Free stall<br>Tie stall
Morabito et al. (2017)	Effects of changing freestall area on lameness, lying time, and leg injuries on dairy farms in Alberta, Canada	Canada	Presence of limp<br>Solano et al. (2015)<br>Vasseur et al. (2015)	839	Free stall
Newsome et al. (2017)	A prospective cohort study of digital cushion and corium thickness. Part 2: Does thinning of the digital cushion and	UK	Score ≥ 2<br>Thomas et al. (2015)	179	Free stall

	corium lead to lameness and claw horn disruption lesions				
O'Driscoll et al. (2009)	The effect of floor surface on dairy cow immune function and locomotion score	Ireland	Not specified, scoring system by O'Callaghan et al. (2003), modified	27	Free stall
Oikonomou et al. (2011)	Effect of polymorphisms at the STAT5A and FGF2 gene loci on reproduction, milk yield and lameness of Holstein cows	Greece	Not specified, scoring system by Sprecher et al. (1997)		Free stall
Onyiro et al. (2008)	Risk factors and milk yield losses associated with lameness in Holstein-Friesian dairy cattle	Scotland	Not specified, scoring system by Manson and Leaver (1988)	248	Free stall
Perez-Cabal and Alenda (2014)	Clinical lameness and risk factors in a Spanish Holstein population	Spain	Gait abnormality	3,459	Free stall
Potzsch et al. (2003)	The impact of parity and duration of biotin supplementation on white line disease lameness in dairy cattle	UK	white line lameness, when examining veterinarian considered that a white line disease was the cause of lameness	900	Free stall
Ristevski et al. (2017a)	Influence of body condition score and ultrasound-determined thickness of body Fat deposit in Holstein-Friesian cows on the risk of lameness developing	Croatia/ Macedonia/ Serbia	Score ≥ 3<br>Sprecher et al. (1997)	100	Free stall
Ristevski et al. (2017b)	Milk production, body condition score and metabolic parameters at the peak of lactation as risk factors for chronic lameness in dairy cows	Croatia/ Macedonia/ Serbia/ Slovenia	Score ≥ 3<br>Sprecher et al. (1997)	100	Free stall
Rouha-Mulleder et al. (2009)	Relative importance of factors influencing the prevalence of lameness in Austrian cubicle loose-housed dairy cows	Austria	Not specified, scoring system by Winckler and Willen (2001)	2,360	Free stall
Sadiq et al. (2017)	Prevalence of lameness, claw lesions, and associated risk factors in dairy farms in Selangor, Malaysia	Malaysia	Score ≥ 3<br>DairyCo (2007)	251	Free stall
Sarjokari et al. (2013)	Prevalence and risk factors for lameness in insulated free stall barns in Finland	Finland	Score ≥ 3<br>Sprecher et al. (1997), modified	3,459	Free stall
Sogstad et al. (2005)	Lameness and claw lesions of the Norwegian red dairy cattle housed in free stalls in relation to environment, parity and stage of lactation	Norway	Not specified	1,540	Free stall
Solano et al. (2015)	Prevalence of lameness and associated risk factors in Canadian Holstein-Friesian cows housed in freestall barns	Canada	Score ≥ 3<br>Flower and Weary (2006)	4981	Free stall
Weber et al. (2013)	Genetic parameters for lameness and claw and leg diseases in dairy cows	Germany	Score ≥ 3<br>Sprecher et al. (1997)	326	Free stall
Wells et al. (1993a)	Individual cow risk factors for clinical lameness in lactating dairy cows	USA	Score ≥ 2 on a 5-point scale (0=none, 1=mild, 2=moderate, 3=severe, 4=non-ambulatory)	1,654	Free stall
Wells et al. (1995a)	Effect of long-term administration of a prolonged release formulation of bovine somatotropin (Sometribove) on clinical lameness in dairy-cows	USA	Score ≥ 2 on a 4-point scale (0=none, 1=mild, 2=moderate, 3=severe)	188	Free stall
Wells et al. (1995b)	Some risk factors associated with clinical lameness in dairy herds in Minnesota and Wisconsin	USA	Score ≥ 2 on a 5-point scale (0=none, 1=mild, 2=moderate, 3=severe, 4=non-ambulatory)	Not available	Free stall<br>Tie stall
Westin et al. (2016b)	Cow- and farm-level risk factors for lameness on dairy farms with automated milking systems	Canada	Presence of obvious limp<br>Flower and Weary (2006)	1,378	Free stall
Wongsanit (2015)	Prevalence and risk factors for lameness in dairy cows raised in small holder farms in western Thailand	Thailand	Score ≥ 3<br>Sprecher et al. (1997)	1,151	Free stall<br>Tie stall

Yaylak et al. (2010)	The effects of several cow and herd level factors on lameness in Holstein cows reared in Izmir Province of Turkey	Turkey	Score ≥ 3<br>Sprecher et al. (1997)	1,078	Free stall

[1]doctoral thesis consisting of four research papers:

Manske, T., J. Hultgren, and C. Bergsten. 2002a. Prevalence and interrelationships of hoof lesions and lameness in Swedish dairy cows. Prev Vet Med. 54:247-263

Manske, T., J. Hultgren, and C. Bergsten. 2002b. A cross-sectional study of risk factors for the hoof health of Swedish dairy cattle. Unpublished

Manske, T., J. Hultgren, and C. Bergsten. 2002c. The effect of claw trimming on the hoof health of Swedish dairy cattle. Prev Vet Med. 54:113-129

Hultgren, J., T. Manske, C. Bergsten. 2004. Associations of sole ulcer at claw trimming with reproductive performance udder health, and culling in Swedish dairy cattle. Prev Vet Med. 62:233-251
