# Supplementary material for: A systematic review and meta-analyses of risk factors associated with lameness in dairy cows
Source: BMC Vet Res. 2019 Oct 16;15:346. doi: 10.1186/s12917-019-2095-2 (PMC6796431; doi:10.1186/s12917-019-2095-2)
Supplement: Supplementary file 4 — Additional file 4. 128 risk factors associated with lameness. [file 12917_2019_2095_MOESM4_ESM.pdf]

Risk factor	Study
Parity	Alban et al., 1996<br>Alban L., 1995<br>Boettcher et al., 1998<br>Dippel et al., 2009a<br>Dippel et al., 2009b<br>Espejo et al., 2006<br>Foditsch et al., 2016<br>Green et al., 2014<br>Groehn et al., 1992<br>Hedges et al., 2001<br>Hultgren et al., 2007<br>King et al., 2017<br>Manske, 2002b<br>Manske, 2002c<br>Morabito et al., 2017<br>Newsome et al., 2017<br>O'Driscoll et al., 2009<br>Potzsch et al., 2003<br>Sadiq et al., 2017<br>Sarjokari et al., 2013<br>Sogstad et al., 2005<br>Solano et al., 2015<br>Weber et al., 2013<br>Wells et al., 1993a<br>Westin et al., 2016b<br>Yaylak et al., 2010
BCS	Becker et al., 2014b<br>Dippel et al., 2009a<br>Dippel et al., 2009b<br>Foditsch et al., 2016<br>Green et al., 2014<br>Gudaj et al., 2012<br>King et al., 2017<br>Morabito et al., 2017<br>Onyiro et al., 2008<br>Ristevski et al., 2017a<br>Ristevski et al., 2017b<br>Sadiq et al., 2017<br>Solano et al., 2015<br>Wells et al., 1993a<br>Westin et al., 2016b<br>Yaylak et al., 2010
DIM	Boettcher et al., 1998<br>Espejo et al., 2006<br>Green et al., 2014<br>Manske, 2002b<br>Manske, 2002c<br>Morabito et al., 2017<br>Pérez-Cabal et al., 2014<br>Sadiq et al., 2017<br>Weber et al., 2013<br>Wells et al., 1993a
Herd size	Adams et al., 2017<br>Alban L., 1995<br>Chapinal et al., 2014<br>Chapinal et al., 2013<br>Dippel et al., 2009b<br>Faye et al., 1989<br>Groehn et al., 1992<br>Solano et al., 2015

	Westin et al., 2016b<br>Yaylak et al., 2010
Flooring surface type (concrete, rubber, etc.)	Adams et al., 2017<br>Faye et al., 1989<br>Frankena et al., 2009<br>Hettich et al., 2007<br>Hultgren et al., 2007<br>Manske, 2002b<br>Manske, 2002c<br>Rouha-Mulleder et al., 2009<br>Wongsanit et al., 2015
Bedding type	Adams et al., 2017<br>Chapinal et al., 2014<br>Chapinal et al., 2013<br>Cook et al., 2016<br>Groehn et al., 1992<br>Pérez-Cabal et al., 2014<br>Rouha-Mulleder et al., 2009
Flooring type (slatted, solid)	Adams et al., 2017<br>Dippel et al., 2009b<br>Frankena et al., 2009<br>Hultgren et al., 2007<br>Pérez-Cabal et al., 2014<br>Rouha-Mulleder et al., 2009<br>Sarjokari et al., 2013
Frequency of claw trimming	Adams et al., 2017<br>Becker et al., 2014b<br>Espejo et al., 2007<br>Manske,2002b<br>Pérez-Cabal et al., 2014<br>Wongsanit et al., 2015
Milk yield	Battagin et al., 2013<br>Green et al., 2014<br>Green et al., 2010<br>Pérez-Cabal et al., 2014<br>Ristevski et al., 2017b<br>Solano et al., 2015
Stall surface/base[1]	Andreasen and Forkman., 2012<br>Cook, 2003<br>Dippel et al., 2009a<br>Dippel et al., 2009b<br>Solano et al., 2015<br>Westin et al., 2016b
Availability of outside exercise area	Adams et al., 2017<br>Becker et al., 2014<br>Dippel et al., 2009b<br>Rouha-Mulleder et al., 2009<br>Wells et al., 1995b
Claw overgrowth	Dembele et al., 2006<br>Morabito et al., 2017<br>Sadiq et al., 2017<br>Solano et al., 2015<br>Wells et al., 1993a
Season	Cook, 2003<br>Foditsch et al., 2016<br>Green et al., 2014<br>Groehn et al., 1992<br>Onyiro et al., 2008
Housing type	Adams et al., 2017<br>Groehn et al., 1992<br>Pérez-Cabal et al., 2014

	Wongsanit et al., 2015
Presence of footbath	Adams et al., 2017<br>Gudaj et al., 2012<br>Hettich et al., 2007<br>Pérez-Cabal et al., 2014
Stall width	Bouffard et al., 2017<br>Dippel et al., 2009b<br>Sogstad et al., 2005<br>Westin et al., 2016b
Access to pasture	Chapinal et al., 2013<br>Pérez-Cabal et al., 2014<br>Wells et al., 1995b
Bedding quantity	Morabito et al., 2017<br>Solano et al., 2015<br>Westin et al., 2016b
Floor slipperiness	Dembele et al., 2006<br>Sarjokari et al., 2013<br>Solano et al., 2015
Frequency of manure removal	Chapinal et al., 2013<br>King et al., 2016<br>Yaylak et al., 2010
General cleanliness	Becker et al., 2014b<br>Dembele et el., 2006<br>Sadiq et al., 2017
Milk protein content	Battagin et al., 2013<br>Dippel et al., 2009b<br>Pérez-Cabal et al., 2014
Neck rail distance to rear curb	Chapinal, 2013<br>Dippel et al., 2009a<br>Rouha-Mulleder et al., 2009
Presence of hock injuries	Morabito et al., 2017<br>Solano et al., 2015<br>Westin et al., 2016b
Breed	Alban L., 1995<br>Sarjokari et al., 2013
Brisket board height	Espejo et al., 2007<br>Westin et al., 2016b
Calving season	Alban et al., 1996<br>Alban L., 1995
Curb height	King et al., 2016<br>Rouha-Mulleder et al., 2009
Length of stalls	Faye et al., 1989<br>Wells et al., 1995b
Lunge space obstruction	Dippel et al., 2009a<br>Westin et al., 2016b
Milk fat content	Battagin et al., 2013<br>Pérez-Cabal et al., 2014
Occurrence of previous lameness	Green et al., 2014<br>Hirst et al., 2002b
Pen area available per cow	Westin et al., 2016b<br>Yaylak et al., 2010
Presence of rubber mats in walkways	Adams et al., 2017<br>Chapinal et al., 2013
Stocking density	King et al., 2016<br>Westin et al., 2016b
Supplementation of biotin	Hedges et al., 2001<br>Potzsch et al., 2003
Temperature	King et al., 2017<br>King et al., 2016
Width of feed alley	Sarjokari et al., 2013<br>Westin et al., 2016b

Abnormal lying behaviour	Dippel et al., 2009b
Angularity	Battagin et al., 2013
Animal keeper is owner	Yaylak et al., 2010
Animal keeper is stockman	Yaylak et al., 2010
Area behind brisket board filled with concrete	Espejo et al., 2007
Availability of feeding expert	Yaylak et al., 2010
Barn age	Chapinal et al., 2014
Bedding cleanliness	Gudaj et al., 2012
Blood BHBA	Ristevski et al., 2017b
Blood LDH	Ristevski et al., 2017b
Blood Triglycerides	Ristevski et al., 2017b
Body weight	Wells et al., 1993a
Breed class	Becker et al., 2014b
Changing diet prior to calving	Hettich et al., 2007
Cleanliness of udder	Becker et al., 2014b
Cleanliness of flank	Becker et al., 2014b
Cleanliness of legs	Westin et al., 2016b
Consideration of claw health in breeding	Becker et al., 2014b
Cow care quality	Dembele et al., 2006
Cow comfort	Espejo et al., 2007
Cow trainer use routine	Alban et al., 1996
Diagnosis season of lameness event	Pérez-Cabal et al., 2014
Distribution of maize silage	Faye et al., 1989
Duration of cow-calf contact	Rouha-Mulleder et al., 2009
Duration of rising process	Dippel et al., 2009b
Farmer's expectation to be farmer in 5 years	Alban L., 1995
Farmer’s opinion of importance of claw health	Becker et al., 2014b
Feed barrier type	Sarjokari et al., 2013
Feeding corn silage in spring	Wells et al., 1995b
Feeding dry concentrate in summer	Wells et al., 1995b
Feeding dry hay	Groehn et al., 1992
Feeding fresh forage in summer	Wells et al., 1995b
Feeding hay in spring	Wells et al., 1995b
Feeding haylage in spring/summer	Wells et al., 1995b
Feeding silage	Becker et al., 2014b
Feeding space available per cow	Westin et al., 2016b
Feeding wet concentrate in summer	Wells et al., 1995b
Free stall care	Morabito et al., 2017
Frequency of footbath	Chapinal et al., 2013
Frequency of raking stalls	Westin et al., 2016b
Frequency of ration balancing	Wells et al., 1995b
Frequency of visits by hoof trimmer	Adams et al., 2017
Gutter width	Wells et al., 1995b
Herd milk yield	Alban et al., 1996
Hock condition score	Sadiq et al., 2017
Hours/day spent in free stalls	Wells et al., 1995b
Hours/day spent in stanchions	Wells et al., 1995b
Hours/day spent in tie stalls	Wells et al., 1995b
Implementation of cow comfort assessment	Morabito et al., 2017
Length of lying area	Dippel et al., 2009b
Long-term administration of sometribove (methionyl bovine somatotropin, 500mg, sc.)	Wells et al., 1995a
Manure removal strategy	Hultgren et al., 2007
Number of cows per milking robot	Westin et al., 2016b
Milking cows that are obviously ill	Gudaj et al., 2012
Number of lactating cows	Wells et al., 1995b
Number of stalls	Gudaj et al., 2012
Percentage of fat cows	Rouha-Mulleder et al., 2009
Percentage of stalls with fecal contamination	Chapinal et al., 2013

Polymorphism at STAT5A and FGF2 gene loci	Oikonomou et al., 2011
Position of neck rail	Bernardi et al., 2009
Position of tie-rail	Bouffard et al., 2017
Presence and condition of stalls with dividers	Wells et al., 1995b
Presence of deep swelling on limbs	Wells et al., 1993a
Presence of fans	Adams et al., 2017
Presence of feed stalls	Hultgren et al., 2007
Presence of infection on limbs	Wells et al., 1993a
Presence of knee injuries	Westin et al., 2016b
Presence of laceration on limbs	Wells et al., 1993a
Presence of skin lesions	Dembele et al., 2006
Presence of sprinklers	Adams et al., 2017
Presence of superficial swelling on limbs	Wells et al., 1993a
Presence of synovial swelling on limbs	Wells et al., 1993a
Previous lactation claw horn disruption lesion-incidence	Foditsch et al., 2016
Promptness of treatment for lameness	Adams et al., 2017
Proportion of cows in cubicles that are lying down	Dippel et al., 2009b
Ratio of concentrate feed to total feed	Yaylak et al., 2010
Rear lateral claw angle	Wells et al., 1993a
Rearing replacement heifers on site	Chapinal et al., 2013
Soil area available per cow	Yaylak et al., 2010
Sole soft tissue thickness	Newsome et al., 2017
Surface moisture	Adams et al., 2017
Thickness of fat over tuber ischiadicum	Ristevski et al., 2017a
Tie rail height	Bouffard et al., 2017
Tie system	Alban et al., 1996
Time away from pen	Espejo et al., 2007
Time of claw trimming	Manske, 2002c
Type of water supply	Sarjokari et al., 2013
Water linear space per cow	Chapinal et al., 2013
Width of entranceway	Wells et al., 1995b
Within-herd prevalence of digital dermatitis and heel-horn erosion	Becker et al., 2014b

[1]The term “stall base” has been used ambiguously. Several studies regarded it as a synonym to “bedding”. Other studies did not follow that interpretation and referred to the composition of the surface beneath the bedding itself. Both terms are displayed separately.
