# Supplementary material for: A systematic review and meta-analyses of risk factors associated with lameness in dairy cows
Source: BMC Vet Res. 2019 Oct 16;15:346. doi: 10.1186/s12917-019-2095-2 (PMC6796431; doi:10.1186/s12917-019-2095-2)
Supplement: Supplementary file 5 — Additional file 5. Implementation of ROBINS-E for the assessment of bias in primary studies . [file 12917_2019_2095_MOESM5_ESM.pdf]

Preliminary tool for risk of bias in exposure studies (1): At protocol stage

Specify the research question by defining a generic target experiment

Participants	*Dairy cows in free stall housing and tie stall facilities*
Experimental exposure	*Set of risk factors associated with lameness*
Control exposure	*Absence of set of risk factors associated with lameness*

List the confounding domains relevant to all or most studies

Breed, milk yield, days in milk/stage of lactation

List the possible co-exposures that could differ between exposure groups and could have an impact on study outcomes

Access to pasture, claw trimming, different housing conditions

List the criteria used to determine the accuracy of exposure measurement

Factors to consider when evaluating health outcome assessment

Preliminary tool for risk of bias in exposure studies (2): For each study

Specify a target experiment specific to the study.

☐	The protocol-specified target experiment fully applies	*OR*	Participant	*9762 dairy cows from 165 Danish dairy herds*
			Experimental exposure	*Free stall housing and tie stall housing exposed to a set of risk factors*
			Control exposure	*Free stall housing and tie stall housing not exposed to a set of risk factors*

Specify the outcome

Specify which outcome is being assessed for risk of bias (typically from among those earmarked for the Summary of Findings table). Specify whether this is a proposed benefit or harm of exposure.

Risk factors of lameness in dairy cows (Possibly benefit and harm of exposure; probably more harm than benefit)

Is your aim for this study…?

☐ to assess the effect of initiating intervention (as in an intention-to-treat analysis)

x to assess the effect of initiating and adhering to intervention (as in a per-protocol analysis)

☐ other (specify)

Specify the numerical result being assessed

In case of multiple alternative analyses being presented, specify the numeric result (e.g. RR = 1.52 (95% CI 0.83 to 2.77) and/or a reference (e.g. to a table, figure or paragraph) that uniquely defines the result being assessed.

Alban L. Lameness in Danish dairy cows - Frequency and possible risk factors. Prev Vet Med. 1995;22:213-25. Table 3

Preliminary consideration of confounders

Complete a row for each important confounding area (i) listed in the review protocol; and (ii) relevant to the setting of this particular study, or which the study authors identified as potentially important.

"Important" confounding areas are those for which, in the context of this study, adjustment is expected to lead to a clinically important change in the estimated effect of the exposure. "Validity" refers to whether the confounding variable or variables fully measure the area, while "reliability" refers to the precision of the measurement (more measurement error means less reliability).

(i) Confounding areas listed in the review protocol

Confounding area	Measured variable(s)	Is there evidence that controlling for this variable was unnecessary?*	Is the confounding area measured validly and reliably by this variable (or these variables)?	OPTIONAL: Is adjusting for this variable (alone) expected to move the effect estimate up or down?
Milk yield	Total milk yield	No	Yes	No information
	Milk yield per day	no		No information
Breed	Breed	No	Yes	No information
Stage of lactation	Days in milk	no		No information

(ii) Additional confounding areas relevant to the setting of this particular study, or which the study authors identified as important

Confounding area	Measured variable(s)	Is there evidence that controlling for this variable was unnecessary?*	Is the confounding area measured validly and reliably by this variable (or these variables)?	OPTIONAL: Is adjusting for this variable (alone) expected to move the effect estimate up or down?

No information provided in the study	No information provided in the study	No information provided in the study	No information provided in the study	No information provided in the study

* In the context of a particular study, variables can be demonstrated not to be confounders and so not included in the analysis: (a) if they are not predictive of the outcome; (b) if they are not predictive of exposure; or (c) because adjustment makes no or minimal difference to the estimated effect of the primary parameter. Note that "no statistically significant association" is not the same as "not predictive".

Preliminary consideration of criteria used to determine the accuracy of measurement of exposure and outcome

Complete a row for each measure listed in the study for the (i) exposure and (ii) outcome. Of the measures listed in the protocol, consider the sensitivity, specificity, and confidence in the methods used in the study.

(i) Exposure measurement method listed in the study		
Method of measurement	Measured exposure	Is the exposure measured validly and reliably by this method (or these methods)?
Central recording of reproductive status, milk yield, disease treatment	Reproductive status, milk yield, disease treatment	No information
Questionnaire about management filled by the farmer	Management practices present on farm	No information

(ii) Outcome measurement method listed in the study		
Method of measurement	Measured outcome	Is the outcome measured validly and reliably by this method (or these methods)?
No information	Lameness in dairy cows defined as	No information

	contusion, foul in the foot sole ulcer, foot rot, interdigital dermatitis, laminits, swollen hock, arthritis, other lameness	

Preliminary consideration of co-exposures

Complete a row for each important co-intervention (i) listed in the review protocol; and (ii) relevant to the setting of this particular study, or which the study authors identified as important.
"Important" co-interventions are those for which, in the context of this study, adjustment is expected to lead to a clinically important change in the estimated effect of the intervention.

**(i) Co-exposures listed in the review protocol**		
Co-exposure	Is there evidence that controlling for this co-exposure was unnecessary (e.g., because it was not administered)?	Is presence of this co-exposure likely to favor outcomes in the experimental or the control group
Access to pasture	No	No information
Claw trimming	Yes	No information
Different housing conditions	No	No information

**(ii) Additional co-exposures relevant to the setting of this particular study, or which the study authors identified as important**

Co-exposure	Is there evidence that controlling for this co-exposure was unnecessary (e.g., because it was not administered)?	Is presence of this co-exposure likely to favor outcomes in the experimental or the control group
No information	No information	No information

Risk of bias assessment (cohort-type studies)

Bias due to confounding	1.1 **Is there potential for confounding of the effect of exposure in this study? If N or PN to 1.1:** the study can be considered to be at low risk of bias due to confounding and no further signaling questions need be considered	Y	
	**If Y/PY to 1.1, answer 2.1 and 1.3 to determine whether there is a need to assess time-varying confounding:**		
	1.2. **If Y or PY to 1.1**: Was the analysis based on splitting follow up time according to exposure received?<br>**If N or PN to 1.2**, answer questions 1.4 to 1.6, which relate to baseline confounding	N	
	1.3. **If Y or PY to 1.2**: Were exposure discontinuations or switches likely to be related to factors that are prognostic for the outcome?	/	
	**If N or PN to 1.3**, answer questions 1.4 to 1.6, which relate to baseline confounding		
	1.4. Did the authors use an appropriate analysis method that adjusted for all the critically important confounding areas?	NI	
	1.5. **If Y or PY to 1.4**: Were confounding areas that were adjusted for measured validly and reliably by the variables available in this study?	NI	
	1.6. Did the authors avoid adjusting for post-exposure variables?	NI	
	**If Y or PY to 1.3**, answer questions 1.7 and 1.8, which relate to time-varying confounding		

	1.7. Did the authors use an appropriate analysis method that adjusted for all the critically important confounding areas and for time-varying confounding?	/	
	1.8. **If Y or PY to 1.7**: Were confounding areas that were adjusted for measured validly and reliably by the variables available in this study?	/	
	**Risk of bias judgement**	Serious	Only scarce information is provided throughout the entire article
	Optional: What is the predicted direction of bias due to confounding?	Unpredictable	Some risk factors may be overestimated whereas other are underestimated and vice-versa
Bias in selection of participants into the study	2.1. Was selection of participants into the study (or into the analysis) based on variables measured after the start of the exposure?<br><br>**If N or PN to 2.1 go to 2.4**	N	
	2.2. **If Y/PY to 2.1:** Were the post-exposure variables that influenced selection associated with exposure?	/	
	2.3. **If Y/PY to 2.2:** Were the post-exposure variables that influenced eligibility selection influenced by the outcome or a cause of the outcome?	/	
	2.4 Do start of follow-up and start of exposure coincide for most participants?	N	
	2.5 If Y/PY to 2.2 and 2.3, or N/PN to 2.4: Were adjustment techniques used that are likely to correct for the presence of selection biases?	NI	
	**Risk of bias judgement**	Moderate	Only partial information is provided throughout the article
	Optional: What is the predicted direction of bias due to selection of participants into the study?	Favors experimental / Favors comparator / Towards null /Away from null / Unpredictable	[Rationale]

Bias in classification of exposures	3.1 Is exposure status well defined?	Y	
	3.2 Did entry into the study begin with start of the exposure?	N	
	3.3 Was information used to define exposure status recorded prior to outcome assessment?	Y	
	3.4 Could classification of exposure status have been affected by knowledge of the outcome or risk of the outcome?	PY	Could potentially have happened. No specific information throughout article
	3.5 Were exposure assessment methods robust (including methods used to input data)?	NI	No information provided on such assessment methods
	**Risk of bias judgement**	Moderate	Only partial information is provided throughout the article
	Optional: What is the predicted direction of bias due to measurement of outcomes or exposures?	Favors experimental / Favors comparator / Towards null /Away from null / Unpredictable	[Rationale]
Bias due to departures from intended exposures	4.1. Is there concern that changes in exposure status occurred among participants?<br><br>**If your aim for this study is to assess the effect of initiating and adhering to an exposure (as in a per-protocol analysis), answer questions 4.2 and 4.3, otherwise continue to 4.4 if Y or PY to 4.1.**	PY	Different cows could potentially have exposed to certain risk factors to a varying extent.
	4.2. Did many participants switch to other exposures?	NI	
	4.3. Were the critical co-exposures balanced across exposure groups?	NI	
	4.4. **If NY/PN PY to 4.1, or Y/PY to 4.2, or 4.3:** Were adjustment techniques used that are likely to correct for these issues?	NI	
	**Risk of bias judgement**	Serious	A serious potential of bias is present as there is scarce information provided

	Optional: What is the predicted direction of bias due to departures from the intended exposures?	Favors experimental / Favors comparator / Towards null /Away from null / Unpredictable	[Rationale]
Bias due to missing data	5.1 Were there missing outcome data?	NI	
	5.2 Were participants excluded due to missing data on exposure status?	Y	
	5.3 Were participants excluded due to missing data on other variables needed for the analysis?	Y	
	5.4 **<u>If Y/PY to 5.1, 5.2 or 5.3:</u>** Are the proportion of participants and reasons for missing data similar across exposures?	NI	
	5.5 **<u>If Y/PY to 5.1, 5.2 or 5.3:</u>** Were appropriate statistical methods used to account for missing data?	NI	
	**Risk of bias judgement**	Serious / Critical / NI	A serious potential of bias is present as there is scarce information provided
	Optional: What is the predicted direction of bias due to missing data?	Favors experimental / Favors comparator / Towards null /Away from null / Unpredictable	[Rationale]
Bias in measurement of outcomes	6.1 Could the outcome measure have been influenced by knowledge of the exposure received?	Y	
	6.2 Was the outcome measure sensitive?	PN	Definition of the outcome variable was vague and covered different conditions
	6.3 Were outcome assessors unaware of the exposure received by study participants?	Y	Data were retrieved without the assessors being involved in data collection
	6.4 Were the methods of outcome assessment comparable across exposure groups?	Y	
	6.5 Were any systematic errors in measurement of the outcome unrelated to exposure received?	NI	

	**Risk of bias judgement**	Moderate	Information only partly available throughout article
	Optional: What is the predicted direction of bias due to measurement of outcomes?	Favors experimental / Favors comparator / Towards null /Away from null / Unpredictable	[Rationale]
Bias in selection of	Is the reported effect estimate likely to be selected, on the basis of the results, from…?		
the reported result	7.1. … multiple outcome *measurements* within the outcome domain?	NI	[Description]
	7.2 … multiple *analyses* of the exposure-outcome relationship?	NI	[Description]
	7.3 … different *subgroups*?	NI	[Description]
	**Risk of bias judgement**	Serious	Potential serious risk since information is lacking throughout article
	Optional: What is the predicted direction of bias due to selection of the reported result?	Favors experimental / Favors comparator / Towards null /Away from null / Unpredictable	[Rationale]
Overall bias	**Risk of bias judgement**	Moderate / Serious	In many parts, information on target questions is not available throughout the article
	Optional:<br>What is the overall predicted direction of bias for this outcome?	Favors experimental / Favors comparator / Towards null /Away from null / Unpredictable	[Rationale]

Preliminary tool for risk of bias in exposure studies (2): For each study

Specify a target experiment specific to the study.

☐	The protocol-specified target experiment fully applies	*OR*	Participant	*1218 dairy cows housed in free stalls*
			Experimental exposure	*A set of risk factors*
			Control exposure	*The absence of this set of risk factors*

Specify the outcome

Specify which outcome is being assessed for risk of bias (typically from among those earmarked for the Summary of Findings table). Specify whether this is a proposed benefit or harm of exposure.

Risk factors of lameness in dairy cows (Possibly benefit and harm of exposure; probably more harm than benefit)

Is your aim for this study...?

☐ to assess the effect of initiating intervention (as in an intention-to-treat analysis)

x to assess the effect of initiating and adhering to intervention (as in a per-protocol analysis)

☐ other (specify)

Specify the numerical result being assessed

In case of multiple alternative analyses being presented, specify the numeric result (e.g. RR = 1.52 (95% CI 0.83 to 2.77) and/or a reference (e.g. to a table, figure or paragraph) that uniquely defines the result being assessed.

King MTM, LeBlanc SJ, Pajor EA, DeVries TJ. Cow-level associations of lameness, behavior, and milk yield of cows milked in automated systems. J Dairy Sci. 2017;100:4818-28. Table 1.

Preliminary consideration of confounders

Complete a row for each important confounding area (i) listed in the review protocol; and (ii) relevant to the setting of this particular study, or which the study authors identified as potentially important.

"Important" confounding areas are those for which, in the context of this study, adjustment is expected to lead to a clinically important change in the estimated effect of the exposure. "Validity" refers to whether the confounding variable or variables fully measure the area, while "reliability" refers to the precision of the measurement (more measurement error means less reliability).

(iii) Confounding areas listed in the review protocol

Confounding area	Measured variable(s)	Is there evidence that controlling for this variable was unnecessary?*	Is the confounding area measured validly and reliably by this variable (or these variables)?	OPTIONAL: Is adjusting for this variable (alone) expected to move the effect estimate up or down?
Milk yield	Total milk yield	No	Yes	No information
	Milk yield per day	no		No information
Breed<br>Stage of lactation	Breed	No	Yes	No information
	Days in milk	no		No information

(iv) Additional confounding areas relevant to the setting of this particular study, or which the study authors identified as important

Confounding area	Measured variable(s)	Is there evidence that controlling for this variable was unnecessary?*	Is the confounding area measured validly and reliably by this variable (or these variables)?	OPTIONAL: Is adjusting for this variable (alone) expected to move the effect estimate up or down?

No information provided in the study	No information provided in the study	No information provided in the study	No information provided in the study	No information provided in the study

* In the context of a particular study, variables can be demonstrated not to be confounders and so not included in the analysis: (a) if they are not predictive of the outcome; (b) if they are not predictive of exposure; or (c) because adjustment makes no or minimal difference to the estimated effect of the primary parameter. Note that "no statistically significant association" is not the same as "not predictive".

Preliminary consideration of criteria used to determine the accuracy of measurement of exposure and outcome

Complete a row for each measure listed in the study for the (i) exposure and (ii) outcome. Of the measures listed in the protocol, consider the sensitivity, specificity, and confidence in the methods used in the study.

(iii) Exposure measurement method listed in the study		
Method of measurement	Measured exposure	Is the exposure measured validly and reliably by this method (or these methods)?
Interview of producers	Routine management practise, feed delivery, feed push-ups, bedding, manure alley management	Partly yes
Recording by researchers	Type of bedding, base material of lying stalls, type of flooring, length of feed bunk, stall dimensions	Yes
Automatic recording by automated milking system	Milk visits<br>Milk related production parameters<br>Parity	Yes

Body condition	Scoring system	Yes

(iv) Outcome measurement method listed in the study		
Method of measurement	Measured outcome	Is the outcome measured validly and reliably by this method (or these methods)?
Locomotion Scoring System	Locomotion	Yes

Preliminary consideration of co-exposures

Complete a row for each important co-intervention (i) listed in the review protocol; and (ii) relevant to the setting of this particular study, or which the study authors identified as important.
"Important" co-interventions are those for which, in the context of this study, adjustment is expected to lead to a clinically important change in the estimated effect of the intervention.

**(iii) Co-exposures listed in the review protocol**		
Co-exposure	Is there evidence that controlling for this co-exposure was unnecessary (e.g., because it was not administered)?	Is presence of this co-exposure likely to favor outcomes in the experimental or the control group
Access to pasture	No information	No information
Claw trimming	No information	No information
Different housing conditions	Yes (all cows in free stall pens)	No information

**(iv) Additional co-exposures relevant to the setting of this particular study, or which the study authors identified as important**

Co-exposure	Is there evidence that controlling for this co-exposure was unnecessary (e.g., because it was not administered)?	Is presence of this co-exposure likely to favor outcomes in the experimental or the control group
No information		No information

Risk of bias assessment (cohort-type studies)

Bias due to confounding	1.1 **Is there potential for confounding of the effect of exposure in this study? If N or PN to 1.1:** the study can be considered to be at low risk of bias due to confounding and no further signaling questions need be considered	PY	No confounders were specified throughout the article
	**If Y/PY to 1.1, answer 2.1 and 1.3 to determine whether there is a need to assess time-varying confounding:**		
	1.2. **If Y or PY to 1.1**: Was the analysis based on splitting follow up time according to exposure received?<br>**If N or PN to 1.2**, answer questions 1.4 to 1.6, which relate to baseline confounding	NI	[Description]
	1.3. **If Y or PY to 1.2**: Were exposure discontinuations or switches likely to be related to factors that are prognostic for the outcome?	NI	
	**If N or PN to 1.3**, answer questions 1.4 to 1.6, which relate to baseline confounding		
	1.4. Did the authors use an appropriate analysis method that adjusted for all the critically important confounding areas?	NI	No information on confounders appears throughout the article
	1.5. **If Y or PY to 1.4**: Were confounding areas that were adjusted for measured validly and reliably by the variables available in this study?	NI	No information on confounders appears throughout the article
	1.6. Did the authors avoid adjusting for post-exposure variables?	NI	No information on confounders appears throughout the article
	**If Y or PY to 1.3**, answer questions 1.7 and 1.8, which relate to time-varying confounding		

	1.7. Did the authors use an appropriate analysis method that adjusted for all the critically important confounding areas and for time-varying confounding?	/	
	1.8. **If Y or PY to 1.7**: Were confounding areas that were adjusted for measured validly and reliably by the variables available in this study?	/	
	**Risk of bias judgement**	Serious /	Potentially serious risk, since no information on confounders was presented throughout the article
	Optional: What is the predicted direction of bias due to confounding?	Favors experimental / Favors comparator / Unpredictable	[Rationale]
Bias in selection of participants into the study	2.1. Was selection of participants into the study (or into the analysis) based on variables measured after the start of the exposure?<br><br>**If N or PN to 2.1 go to 2.4**	N	
	2.2. **If Y/PY to 2.1:** Were the post-exposure variables that influenced selection associated with exposure?	/	
	2.3. **If Y/PY to 2.2:** Were the post-exposure variables that influenced eligibility selection influenced by the outcome or a cause of the outcome?	/	
	2.4 Do start of follow-up and start of exposure coincide for most participants?	N	
	2.5 If Y/PY to 2.2 and 2.3, or N/PN to 2.4: Were adjustment techniques used that are likely to correct for the presence of selection biases?	/	/
	**Risk of bias judgement**	Low	Selection of participants was performed before animal based data were collected
	Optional: What is the predicted direction of bias due to selection of participants into the study?	Favors experimental / Favors comparator / Towards null	[Rationale]

		/Away from null / Unpredictable	
Bias in classification of exposures	3.1 Is exposure status well defined?	Y	
	3.2 Did entry into the study begin with start of the exposure?	N	
	3.3 Was information used to define exposure status recorded prior to outcome assessment?	NI	
	3.4 Could classification of exposure status have been affected by knowledge of the outcome or risk of the outcome?	PY	The collecting of some data could potentially have been influenced by knowledge of the outcome
	3.5 Were exposure assessment methods robust (including methods used to input data)?	Y	
	**Risk of bias judgement**	Low/moderate	Potentially low to moderate risk of bias, since entry into the study was after start of exposure: However, some measure could have been influenced during data collection against the background of knowledge of the outcome
	Optional: What is the predicted direction of bias due to measurement of outcomes or exposures?	Favors experimental / Favors comparator / Towards null /Away from null / Unpredictable	[Rationale]
Bias due to departures from intended exposures	4.1. Is there concern that changes in exposure status occurred among participants?<br><br>**If your aim for this study is to assess the effect of initiating and adhering to an exposure (as in a per-protocol analysis), answer questions 4.2 and 4.3, otherwise continue to 4.4 if Y or PY to 4.1.**	NI	
	4.2. Did many participants switch to other exposures?	NI	

	4.3. Were the critical co-exposures balanced across exposure groups?	NI	
	4.4. **<u>If NY/PN PY to 4.1, or Y/PY to 4.2, or 4.3:</u>** Were adjustment techniques used that are likely to correct for these issues?	/	
	**Risk of bias judgement**	Serious	Potentially serious risk of bias, since information is not provided
	Optional: What is the predicted direction of bias due to departures from the intended exposures?	Favors experimental / Favors comparator / Towards null /Away from null / Unpredictable	[Rationale]
Bias due to missing data	5.1 Were there missing outcome data?	Y	[Description]
	5.2 Were participants excluded due to missing data on exposure status?	Y	[Description]
	5.3 Were participants excluded due to missing data on other variables needed for the analysis?	Y	[Description]
	5.4 **<u>If Y/PY to 5.1, 5.2 or 5.3:</u>** Are the proportion of participants and reasons for missing data similar across exposures?	NI	[Description]
	5.5 **<u>If Y/PY to 5.1, 5.2 or 5.3:</u>** Were appropriate statistical methods used to account for missing data?	NI	[Description]
	**Risk of bias judgement**	Moderate / Serious	Potentially moderate to serious risk of bias since information is partly not available
	Optional: What is the predicted direction of bias due to missing data?	Favors experimental / Favors comparator / Towards null /Away from null / Unpredictable	[Rationale]
Bias in measurement of outcomes	6.1 Could the outcome measure have been influenced by knowledge of the exposure received?	Y	[Description]
	6.2 Was the outcome measure sensitive?	PY	Locomotion scoring is rather sensitive, however subjective
	6.3 Were outcome assessors unaware of the exposure received by study participants?	NI	[Description]

	6.4 Were the methods of outcome assessment comparable across exposure groups?	Y	[Description]
	6.5 Were any systematic errors in measurement of the outcome unrelated to exposure received?	NI	[Description]
	**Risk of bias judgement**	Moderate / Serious	Moderate to serious risk of bias since measures could have been influenced by knowledge of outcome and partially information is not available
	Optional: What is the predicted direction of bias due to measurement of outcomes?	Favors experimental / Favors comparator / Towards null /Away from null / Unpredictable	[Rationale]
Bias in selection of	Is the reported effect estimate likely to be selected, on the basis of the results, from...?		
the reported result	7.1. ... multiple outcome *measurements* within the outcome domain?	NI	[Description]
	7.2 ... multiple *analyses* of the exposure-outcome relationship?	NI	[Description]
	7.3 ... different *subgroups*?	NI	[Description]
	**Risk of bias judgement**	NI	Potentially serious. However no information available
	Optional: What is the predicted direction of bias due to selection of the reported result?	Favors experimental / Favors comparator / Towards null /Away from null / Unpredictable	[Rationale]
Overall bias	**Risk of bias judgement**	Moderate / Serious	In many parts, bias could have entered this work. This is intensified by the fact that information is scarce
	Optional:<br>What is the overall predicted direction of bias for this outcome?	Favors experimental / Favors comparator / Towards null /Away from null / Unpredictable	[Rationale]

Preliminary tool for risk of bias in exposure studies (2): For each study

Specify a target experiment specific to the study.

☐	The protocol-specified target experiment fully applies	*OR*	Participant	*4,899/3,444/2,368 dairy cows*
			Experimental exposure	*Free stall housing and tie stall housing exposed to a set of risk factors*
			Control exposure	*Free stall housing and tie stall housing not exposed to a set of risk factors*

Specify the outcome

Specify which outcome is being assessed for risk of bias (typically from among those earmarked for the Summary of Findings table). Specify whether this is a proposed benefit or harm of exposure.

Risk factors of lameness in dairy cows (Possibly benefit and harm of exposure; probably more harm than benefit)

Is your aim for this study…?

☐ to assess the effect of initiating intervention (as in an intention-to-treat analysis)

x to assess the effect of initiating and adhering to intervention (as in a per-protocol analysis)

☐ other (specify)

Specify the numerical result being assessed

In case of multiple alternative analyses being presented, specify the numeric result (e.g. RR = 1.52 (95% CI 0.83 to 2.77) and/or a reference (e.g. to a table, figure or paragraph) that uniquely defines the result being assessed.

Manske T. Hoof lesions and lameness in Swedish dairy cattle : prevalence, risk factors, effects of claw trimming, and consequences for productivity. PhD thesis. Acta Universitatis Agriculturae Sueciae 135: Skara : Swedish University of Agricultural Sciences, 2002. Table I in Paper I.

Preliminary consideration of confounders

Complete a row for each important confounding area (i) listed in the review protocol; and (ii) relevant to the setting of this particular study, or which the study authors identified as potentially important.
"Important" confounding areas are those for which, in the context of this study, adjustment is expected to lead to a clinically important change in the estimated effect of the exposure. "Validity" refers to whether the confounding variable or variables fully measure the area, while "reliability" refers to the precision of the measurement (more measurement error means less reliability).

**(v) Confounding areas listed in the review protocol**				
Confounding area	Measured variable(s)	Is there evidence that controlling for this variable was unnecessary?*	Is the confounding area measured validly and reliably by this variable (or these variables)?	OPTIONAL: Is adjusting for this variable (alone) expected to move the effect estimate up or down?
Milk yield	Total milk yield	No	Yes	No information
	Milk yield per day	no		No information
Breed<br>Stage of lactation	Breed	No	Yes	No information
	Days in milk	no		No information

**(vi) Additional confounding areas relevant to the setting of this particular study, or which the study authors identified as important**				
Confounding area	Measured variable(s)	Is there evidence that controlling for this variable was unnecessary?*	Is the confounding area measured validly and reliably by this variable (or these variables)?	OPTIONAL: Is adjusting for this variable (alone) expected to move the effect estimate up or down?

No information provided in the study	No information provided in the study	No information provided in the study	No information provided in the study	No information provided in the study

* In the context of a particular study, variables can be demonstrated not to be confounders and so not included in the analysis: (a) if they are not predictive of the outcome; (b) if they are not predictive of exposure; or (c) because adjustment makes no or minimal difference to the estimated effect of the primary parameter. Note that "no statistically significant association" is not the same as "not predictive".

Preliminary consideration of criteria used to determine the accuracy of measurement of exposure and outcome

Complete a row for each measure listed in the study for the (i) exposure and (ii) outcome. Of the measures listed in the protocol, consider the sensitivity, specificity, and confidence in the methods used in the study.

(v) Exposure measurement method listed in the study		
Method of measurement	Measured exposure	Is the exposure measured validly and reliably by this method (or these methods)?
Official milk-recording scheme	Breed, parity, calving date	No information
Special visits to herds	Housing system, feeding routines, management	yes
Measurements	Building measurements, temperature, humidity	yes
Scoring	Dampness of lying surface, abrasiveness of floors level of air-ammonium	Partly yes (subjective scoring)
Interview with farmer	Previous hoof trimming history, heifer rearing, feeding routines, amount og bedding, etc.)	Partly yes (possible qualitative interaction between observer and observed)

(vi) Outcome measurement method listed in the study

Method of measurement	Measured outcome	Is the outcome measured validly and reliably by this method (or these methods)?
Scoring on ordinal scale, then dichotomization	Lameness	No information on exact procedure and criteria of scoring

Preliminary consideration of co-exposures

Complete a row for each important co-intervention (i) listed in the review protocol; and (ii) relevant to the setting of this particular study, or which the study authors identified as important.
"Important" co-interventions are those for which, in the context of this study, adjustment is expected to lead to a clinically important change in the estimated effect of the intervention.

**(v) Co-exposures listed in the review protocol**		
Co-exposure	Is there evidence that controlling for this co-exposure was unnecessary (e.g., because it was not administered)?	Is presence of this co-exposure likely to favor outcomes in the experimental or the control group
Access to pasture	No	No information
Claw trimming	Yes	Yes
Different housing conditions	Yes	No information

**(vi) Additional co-exposures relevant to the setting of this particular study, or which the study authors identified as important**		
Co-exposure	Is there evidence that controlling for this co-exposure was unnecessary (e.g., because it was not administered)?	Is presence of this co-exposure likely to favor outcomes in the experimental or the control group
No information	No information	No information

Risk of bias assessment (cohort-type studies)

Bias due to confounding	1.1 **Is there potential for confounding of the effect of exposure in this study? If N or PN to 1.1:** the study can be considered to be at low risk of bias due to confounding and no further signaling questions need be considered	Y	
	**If Y/PY to 1.1, answer 2.1 and 1.3 to determine whether there is a need to assess time-varying confounding:**		
	1.2. **If Y or PY to 1.1**: Was the analysis based on splitting follow up time according to exposure received?<br>**If N or PN to 1.2**, answer questions 1.4 to 1.6, which relate to baseline confounding	NI	
	1.3. **If Y or PY to 1.2**: Were exposure discontinuations or switches likely to be related to factors that are prognostic for the outcome?	/	
	**If N or PN to 1.3**, answer questions 1.4 to 1.6, which relate to baseline confounding		
	1.4. Did the authors use an appropriate analysis method that adjusted for all the critically important confounding areas?	NI	
	1.5. **If Y or PY to 1.4**: Were confounding areas that were adjusted for measured validly and reliably by the variables available in this study?	NI	
	1.6. Did the authors avoid adjusting for post-exposure variables?	NI	
	**If Y or PY to 1.3**, answer questions 1.7 and 1.8, which relate to time-varying confounding		

	1.7. Did the authors use an appropriate analysis method that adjusted for all the critically important confounding areas and for time-varying confounding?	/	
	1.8. **If Y or PY to 1.7**: Were confounding areas that were adjusted for measured validly and reliably by the variables available in this study?	/	
	**Risk of bias judgement**	Serious	Only scarce information is provided throughout the entire work
	Optional: What is the predicted direction of bias due to confounding?	/	
Bias in selection of participants into the study	2.1. Was selection of participants into the study (or into the analysis) based on variables measured after the start of the exposure?<br><br>**If N or PN to 2.1 go to 2.4**	PY	Some information created a risk of qualitative and quantitative interaction between the observer and the observed. Allocation to one treatment group was done after possible exclusion
	2.2. **If Y/PY to 2.1:** Were the post-exposure variables that influenced selection associated with exposure?	PY	
	2.3. **If Y/PY to 2.2:** Were the post-exposure variables that influenced eligibility selection influenced by the outcome or a cause of the outcome?	PY	
	2.4 Do start of follow-up and start of exposure coincide for most participants?	NI	
	2.5 If Y/PY to 2.2 and 2.3, or N/PN to 2.4: Were adjustment techniques used that are likely to correct for the presence of selection biases?	NI	
	**Risk of bias judgement**	Serious	Potentially serious risk since some contamination and bias potentially entered study
	Optional: What is the predicted direction of bias due to selection of participants into the study?	Favors experimental / Favors comparator / Towards null	[Rationale]

		/Away from null / Unpredictable	
Bias in classification of exposures	3.1 Is exposure status well defined?	Y	
	3.2 Did entry into the study begin with start of the exposure?	PY	Some information created a risk of qualitative and quantitative interaction between the observer and the observed. In some cases exposure may have started after beginning
	3.3 Was information used to define exposure status recorded prior to outcome assessment?	NI	
	3.4 Could classification of exposure status have been affected by knowledge of the outcome or risk of the outcome?	PY	Could potentially have happened.
	3.5 Were exposure assessment methods robust (including methods used to input data)?	PY	
	**Risk of bias judgement**	Moderate	Risk of entry of bias at several levels of the work. However addressed throughout work
	Optional: What is the predicted direction of bias due to measurement of outcomes or exposures?	Favors experimental / Favors comparator / Towards null /Away from null / Unpredictable	[Rationale]
Bias due to departures from intended exposures	4.1. Is there concern that changes in exposure status occurred among participants?<br><br>**If your aim for this study is to assess the effect of initiating and adhering to an exposure (as in a per-protocol analysis), answer questions 4.2 and 4.3, otherwise continue to 4.4 if Y or PY to 4.1.**	PY	Possible since exposure could have been influenced
	4.2. Did many participants switch to other exposures?	NI	
	4.3. Were the critical co-exposures balanced across exposure groups?	NI	

	4.4. **<u>If NY/PN PY to 4.1, or Y/PY to 4.2, or 4.3:</u>** Were adjustment techniques used that are likely to correct for these issues?	NI	
	**Risk of bias judgement**	Serious	A serious potential of bias is present as there is scarce information provided and because exposure could have changed
	Optional: What is the predicted direction of bias due to departures from the intended exposures?	Favors experimental / Favors comparator / Towards null /Away from null / Unpredictable	[Rationale]
Bias due to missing data	5.1 Were there missing outcome data?	NI	
	5.2 Were participants excluded due to missing data on exposure status?	NI	
	5.3 Were participants excluded due to missing data on other variables needed for the analysis?	NI	
	5.4 **<u>If Y/PY to 5.1, 5.2 or 5.3:</u>** Are the proportion of participants and reasons for missing data similar across exposures?	NI	
	5.5 **<u>If Y/PY to 5.1, 5.2 or 5.3:</u>** Were appropriate statistical methods used to account for missing data?	NI	
	**Risk of bias judgement**	Serious	A serious potential of bias is present as there is scarce information provided
	Optional: What is the predicted direction of bias due to missing data?	Favors experimental / Favors comparator / Towards null /Away from null / Unpredictable	[Rationale]
Bias in measurement of outcomes	6.1 Could the outcome measure have been influenced by knowledge of the exposure received?	Y	
	6.2 Was the outcome measure sensitive?	PN	Outcome variable was assessed by subjective scoring
	6.3 Were outcome assessors unaware of the exposure received by study participants?	PN	Data were retrieved and observers could have influenced the observed

	6.4 Were the methods of outcome assessment comparable across exposure groups?	Y	
	6.5 Were any systematic errors in measurement of the outcome unrelated to exposure received?	NI	
	**Risk of bias judgement**	Serious	Information only partly available throughout article. Furthermore The exposure received could have been influenced knowing the outcome
	Optional: What is the predicted direction of bias due to measurement of outcomes?	Favors experimental / Favors comparator / Towards null /Away from null / Unpredictable	[Rationale]
Bias in selection of	Is the reported effect estimate likely to be selected, on the basis of the results, from...?		
the reported result	7.1. ... multiple outcome *measurements* within the outcome domain?	NI	[Description]
	7.2 ... multiple *analyses* of the exposure-outcome relationship?	NI	[Description]
	7.3 ... different *subgroups*?	NI	[Description]
	**Risk of bias judgement**	Serious	Potential serious risk since information is lacking throughout article
	Optional: What is the predicted direction of bias due to selection of the reported result?	Favors experimental / Favors comparator / Towards null /Away from null / Unpredictable	[Rationale]
Overall bias	**Risk of bias judgement**	Moderate / Serious	In many parts, information on target questions is not available throughout the article
	Optional:<br>What is the overall predicted direction of bias for this outcome?	Favors experimental / Favors comparator / Towards null /Away from null / Unpredictable	[Rationale]

Preliminary tool for risk of bias in exposure studies (2): For each study

Specify a target experiment specific to the study.

☐ The protocol-specified target experiment fully applies *OR*

Participant	*251 dairy cows*
Experimental exposure	*Free stall housing exposed to a set of risk factors*
Control exposure	*Free stall housing not exposed to a set of risk factors*

Specify the outcome

Specify which outcome is being assessed for risk of bias (typically from among those earmarked for the Summary of Findings table). Specify whether this is a proposed benefit or harm of exposure.

Risk factors of lameness in dairy cows (Possibly benefit and harm of exposure; probably more harm than benefit)

Is your aim for this study…?

☐ to assess the effect of initiating intervention (as in an intention-to-treat analysis)

x to assess the effect of initiating and adhering to intervention (as in a per-protocol analysis)

☐ other (specify)

Specify the numerical result being assessed

In case of multiple alternative analyses being presented, specify the numeric result (e.g. RR = 1.52 (95% CI 0.83 to 2.77) and/or a reference (e.g. to a table, figure or paragraph) that uniquely defines the result being assessed.

Sadiq MB, Ramanoon SZ, Mansor R, Syed-Hussain SS, Mossadeq WMS. Prevalence of lameness, claw lesions, and associated risk factors in dairy farms in Selangor, Malaysia. Trop Anim Health Prod. 2017;49:1741-8. Table 4

Preliminary consideration of confounders

Complete a row for each important confounding area (i) listed in the review protocol; and (ii) relevant to the setting of this particular study, or which the study authors identified as potentially important.
"Important" confounding areas are those for which, in the context of this study, adjustment is expected to lead to a clinically important change in the estimated effect of the exposure. "Validity" refers to whether the confounding variable or variables fully measure the area, while "reliability" refers to the precision of the measurement (more measurement error means less reliability).

(vii) Confounding areas listed in the review protocol

Confounding area	Measured variable(s)	Is there evidence that controlling for this variable was unnecessary?*	Is the confounding area measured validly and reliably by this variable (or these variables)?	OPTIONAL: Is adjusting for this variable (alone) expected to move the effect estimate up or down?
Milk yield	Total milk yield	No	Yes	No information
	Milk yield per day	no		No information
Breed<br>Stage of lactation	Breed	No	Yes	No information
	Days in milk	no		No information

(viii) **Additional confounding areas relevant to the setting of this particular study, or which the study authors identified as important**

Confounding area	Measured variable(s)	Is there evidence that controlling for this variable was unnecessary?*	Is the confounding area measured validly and reliably by this variable (or these variables)?	OPTIONAL: Is adjusting for this variable (alone) expected to move the effect estimate up or down?

No information provided in the study	No information provided in the study	No information provided in the study	No information provided in the study	No information provided in the study

* In the context of a particular study, variables can be demonstrated not to be confounders and so not included in the analysis: (a) if they are not predictive of the outcome; (b) if they are not predictive of exposure; or (c) because adjustment makes no or minimal difference to the estimated effect of the primary parameter. Note that “no statistically significant association” is not the same as “not predictive”.

Preliminary consideration of criteria used to determine the accuracy of measurement of exposure and outcome

Complete a row for each measure listed in the study for the (i) exposure and (ii) outcome. Of the measures listed in the protocol, consider the sensitivity, specificity, and confidence in the methods used in the study.

(vii) Exposure measurement method listed in the study		
Method of measurement	Measured exposure	Is the exposure measured validly and reliably by this method (or these methods)?
Recording before assessing locomotion	Body condition score, hock condition score, leg hygiene	Partly yes (subjective scoring system)
Farm records and self-administered questionnaire	Herd size, number of milking cows, number of cows at early days in milk, access to pasture	No information

(viii) Outcome measurement method listed in the study		
Method of measurement	Measured outcome	Is the outcome measured validly and reliably by this method (or these methods)?
Scoring system	Lameness	Partly yes (subjective scoring system)

Preliminary consideration of co-exposures

Complete a row for each important co-intervention (i) listed in the review protocol; and (ii) relevant to the setting of this particular study, or which the study authors identified as important.
"Important" co-interventions are those for which, in the context of this study, adjustment is expected to lead to a clinically important change in the estimated effect of the intervention.

**(vii) Co-exposures listed in the review protocol**		
Co-exposure	Is there evidence that controlling for this co-exposure was unnecessary (e.g., because it was not administered)?	Is presence of this co-exposure likely to favor outcomes in the experimental or the control group
Access to pasture	No	No information
Claw trimming	No	No information
Different housing conditions	No	No information

**(viii) Additional co-exposures relevant to the setting of this particular study, or which the study authors identified as important**		
Co-exposure	Is there evidence that controlling for this co-exposure was unnecessary (e.g., because it was not administered)?	Is presence of this co-exposure likely to favor outcomes in the experimental or the control group
No information	No information	No information

Risk of bias assessment (cohort-type studies)

Bias due to confounding	1.1 **Is there potential for confounding of the effect of exposure in this study? If N or PN to 1.1:** the study can be considered to be at low risk of bias due to confounding and no further signaling questions need be considered	Y	
	**If Y/PY to 1.1, answer 2.1 and 1.3 to determine whether there is a need to assess time-varying confounding:**		
	1.2. <u>**If Y or PY to 1.1**</u>: Was the analysis based on splitting follow up time according to exposure received?<br>**If N or PN to 1.2**, answer questions 1.4 to 1.6, which relate to baseline confounding	NI	
	1.3. **If Y or PY to 1.2**: Were exposure discontinuations or switches likely to be related to factors that are prognostic for the outcome?	/	
	**If N or PN to 1.3**, answer questions 1.4 to 1.6, which relate to baseline confounding		
	1.4. Did the authors use an appropriate analysis method that adjusted for all the critically important confounding areas?	NI	
	1.5. **If Y or PY to 1.4**: Were confounding areas that were adjusted for measured validly and reliably by the variables available in this study?	NI	
	1.6. Did the authors avoid adjusting for post-exposure variables?	NI	
	**If Y or PY to 1.3**, answer questions 1.7 and 1.8, which relate to time-varying confounding		

	1.7. Did the authors use an appropriate analysis method that adjusted for all the critically important confounding areas and for time-varying confounding?	/	
	1.8. **If Y or PY to 1.7**: Were confounding areas that were adjusted for measured validly and reliably by the variables available in this study?	/	
	**Risk of bias judgement**	Serious	Information is hardly provided throughout the entire article
	Optional: What is the predicted direction of bias due to confounding?	/	
Bias in selection of participants into the study	2.1. Was selection of participants into the study (or into the analysis) based on variables measured after the start of the exposure?<br><br>**<u>If N or PN to 2.1 go to 2.4</u>**	N	
	2.2. **<u>If Y/PY to 2.1:</u>** Were the post-exposure variables that influenced selection associated with exposure?	/	
	2.3. **<u>If Y/PY to 2.2:</u>** Were the post-exposure variables that influenced eligibility selection influenced by the outcome or a cause of the outcome?	/	
	2.4 Do start of follow-up and start of exposure coincide for most participants?	N	
	2.5 If Y/PY to 2.2 and 2.3, or N/PN to 2.4: Were adjustment techniques used that are likely to correct for the presence of selection biases?	NI	
	**Risk of bias judgement**	Moderate	Only partial information is provided throughout the article
	Optional: What is the predicted direction of bias due to selection of participants into the study?	Favors experimental / Favors comparator / Towards null /Away from null / Unpredictable	[Rationale]

Bias in classification of exposures	3.1 Is exposure status well defined?	NI	
	3.2 Did entry into the study begin with start of the exposure?	N	
	3.3 Was information used to define exposure status recorded prior to outcome assessment?	NI	
	3.4 Could classification of exposure status have been affected by knowledge of the outcome or risk of the outcome?	PY	Could potentially have happened. No specific information throughout article
	3.5 Were exposure assessment methods robust (including methods used to input data)?	PY	Not much information provided on such assessment methods. Implementation of subjective scoring methods.
	**Risk of bias judgement**	Serious	Only very scarce information is provided throughout the article
	Optional: What is the predicted direction of bias due to measurement of outcomes or exposures?	Favors experimental / Favors comparator / Towards null /Away from null / Unpredictable	[Rationale]
Bias due to departures from intended exposures	4.1. Is there concern that changes in exposure status occurred among participants?<br><br>**If your aim for this study is to assess the effect of initiating and adhering to an exposure (as in a per-protocol analysis), answer questions 4.2 and 4.3, otherwise continue to 4.4 if Y or PY to 4.1.**	NI	
	4.2. Did many participants switch to other exposures?	NI	
	4.3. Were the critical co-exposures balanced across exposure groups?	NI	
	4.4. <u>**If NY/PN PY to 4.1, or Y/PY to 4.2, or 4.3:**</u> Were adjustment techniques used that are likely to correct for these issues?	NI	

	**Risk of bias judgement**	Serious	A serious potential of bias is present as there is hardly any information provided
	Optional: What is the predicted direction of bias due to departures from the intended exposures?	Favors experimental / Favors comparator / Towards null /Away from null / Unpredictable	[Rationale]
Bias due to missing data	5.1 Were there missing outcome data?	NI	
	5.2 Were participants excluded due to missing data on exposure status?	NI	
	5.3 Were participants excluded due to missing data on other variables needed for the analysis?	NI	
	5.4 **<u>If Y/PY to 5.1, 5.2 or 5.3:</u>** Are the proportion of participants and reasons for missing data similar across exposures?	NI	
	5.5 **<u>If Y/PY to 5.1, 5.2 or 5.3:</u>** Were appropriate statistical methods used to account for missing data?	NI	
	**Risk of bias judgement**	Serious	A serious potential of bias is present as there is scarce information provided
	Optional: What is the predicted direction of bias due to missing data?	Favors experimental / Favors comparator / Towards null /Away from null / Unpredictable	[Rationale]
Bias in measurement of outcomes	6.1 Could the outcome measure have been influenced by knowledge of the exposure received?	Y	
	6.2 Was the outcome measure sensitive?	PN	Subjective assessment of outcome variable via subjective scoring system
	6.3 Were outcome assessors unaware of the exposure received by study participants?	N	
	6.4 Were the methods of outcome assessment comparable across exposure groups?	Y	
	6.5 Were any systematic errors in measurement of the outcome unrelated to exposure received?	NI	

	**Risk of bias judgement**	Serious	Little information available. Knowledge of outcome could have influenced assessments
	Optional: What is the predicted direction of bias due to measurement of outcomes?	Favors experimental / Favors comparator / Towards null /Away from null / Unpredictable	[Rationale]
Bias in selection of	Is the reported effect estimate likely to be selected, on the basis of the results, from...?		
the reported result	7.1. ... multiple outcome *measurements* within the outcome domain?	NI	[Description]
	7.2 ... multiple *analyses* of the exposure-outcome relationship?	NI	[Description]
	7.3 ... different *subgroups*?	NI	[Description]
	**Risk of bias judgement**	Serious	Potential serious risk since information is lacking throughout article
	Optional: What is the predicted direction of bias due to selection of the reported result?	Favors experimental / Favors comparator / Towards null /Away from null / Unpredictable	[Rationale]
Overall bias	**Risk of bias judgement**	Serious	In many parts, information on target questions is not available throughout the article
	Optional:<br>What is the overall predicted direction of bias for this outcome?	Favors experimental / Favors comparator / Towards null /Away from null / Unpredictable	[Rationale]

Preliminary tool for risk of bias in exposure studies (2): For each study

Specify a target experiment specific to the study.

☐	The protocol-specified target experiment fully applies	*OR*	Participant	*4981 dairy cows*
			Experimental exposure	*Free stall housing and a set of risk factors*
			Control exposure	*Free stall housing not exposed to a set of risk factors*

Specify the outcome

Specify which outcome is being assessed for risk of bias (typically from among those earmarked for the Summary of Findings table). Specify whether this is a proposed benefit or harm of exposure.

Risk factors of lameness in dairy cows (Possibly benefit and harm of exposure; probably more harm than benefit)

Is your aim for this study…?

☐ to assess the effect of initiating intervention (as in an intention-to-treat analysis)

x to assess the effect of initiating and adhering to intervention (as in a per-protocol analysis)

☐ other (specify)

Specify the numerical result being assessed

In case of multiple alternative analyses being presented, specify the numeric result (e.g. RR = 1.52 (95% CI 0.83 to 2.77) and/or a reference (e.g. to a table, figure or paragraph) that uniquely defines the result being assessed.

Solano L, Barkema HW, Pajor EA, Mason S, LeBlanc SJ, Heyerhoff JCZ, et al. Prevalence of lameness and associated risk factors in Canadian Holstein-Friesian cows housed in freestall barns. J Dairy Sci. 2015;98:6978-91.

Preliminary consideration of confounders

Complete a row for each important confounding area (i) listed in the review protocol; and (ii) relevant to the setting of this particular study, or which the study authors identified as potentially important.
"Important" confounding areas are those for which, in the context of this study, adjustment is expected to lead to a clinically important change in the estimated effect of the exposure. "Validity" refers to whether the confounding variable or variables fully measure the area, while "reliability" refers to the precision of the measurement (more measurement error means less reliability).

**(ix) Confounding areas listed in the review protocol**				
Confounding area	Measured variable(s)	Is there evidence that controlling for this variable was unnecessary?*	Is the confounding area measured validly and reliably by this variable (or these variables)?	OPTIONAL: Is adjusting for this variable (alone) expected to move the effect estimate up or down?
Milk yield	Total milk yield	No	Yes	No information
	Milk yield per day	no		No information
Breed<br>Stage of lactation	Breed	No	Yes	No information
	Days in milk	no		No information

**(x) Additional confounding areas relevant to the setting of this particular study, or which the study authors identified as important**				
Confounding area	Measured variable(s)	Is there evidence that controlling for this variable was unnecessary?*	Is the confounding area measured validly and reliably by this variable (or these variables)?	OPTIONAL: Is adjusting for this variable (alone) expected to move the effect estimate up or down?

No information provided in the study	No information provided in the study	No information provided in the study	No information provided in the study	No information provided in the study

* In the context of a particular study, variables can be demonstrated not to be confounders and so not included in the analysis: (a) if they are not predictive of the outcome; (b) if they are not predictive of exposure; or (c) because adjustment makes no or minimal difference to the estimated effect of the primary parameter. Note that "no statistically significant association" is not the same as "not predictive".

Preliminary consideration of criteria used to determine the accuracy of measurement of exposure and outcome

Complete a row for each measure listed in the study for the (i) exposure and (ii) outcome. Of the measures listed in the protocol, consider the sensitivity, specificity, and confidence in the methods used in the study.

(ix) Exposure measurement method listed in the study		
Method of measurement	Measured exposure	Is the exposure measured validly and reliably by this method (or these methods)?
Scoring	Leg cleanliness, BCS, hock injuries, claw length	Partly yes (subjective scoring system)
Questionnaire/interview	General management	Partly yes. Possibly subjectively influenced
Assessment/measuring	Type of flooring, width of feed alley, floor cleanliness, floor slipperiness	Partly yes
Assessment/measuring	Stocking density, stall dimensions, stall base, stall bedding type, cleanliness, quantity, dryness, foot bath	Partly yes

(x) Outcome measurement method listed in the study		
Method of measurement	Measured outcome	Is the outcome measured validly and reliably by this method (or these methods)?
No information		No information
Numerical rating score	Lameness	Partly yes (subjective scoring system)

Preliminary consideration of co-exposures

Complete a row for each important co-intervention (i) listed in the review protocol; and (ii) relevant to the setting of this particular study, or which the study authors identified as important.
"Important" co-interventions are those for which, in the context of this study, adjustment is expected to lead to a clinically important change in the estimated effect of the intervention.

**(ix) Co-exposures listed in the review protocol**		
Co-exposure	Is there evidence that controlling for this co-exposure was unnecessary (e.g., because it was not administered)?	Is presence of this co-exposure likely to favor outcomes in the experimental or the control group
Access to pasture	Yes	No information
Claw trimming	No	No information
Different housing conditions	Yes	No information

**(x) Additional co-exposures relevant to the setting of this particular study, or which the study authors identified as important**		
Co-exposure	Is there evidence that controlling for this co-exposure was unnecessary (e.g., because it was not administered)?	Is presence of this co-exposure likely to favor outcomes in the experimental or the control group

No information	No information	No information

Risk of bias assessment (cohort-type studies)

Bias due to confounding	1.1 **Is there potential for confounding of the effect of exposure in this study? If N or PN to 1.1:** the study can be considered to be at low risk of bias due to confounding and no further signaling questions need be considered	Y	
	**If Y/PY to 1.1, answer 2.1 and 1.3 to determine whether there is a need to assess time-varying confounding:**		
	1.2. **If Y or PY to 1.1:** Was the analysis based on splitting follow up time according to exposure received?<br>**If N or PN to 1.2**, answer questions 1.4 to 1.6, which relate to baseline confounding	N	
	1.3. **If Y or PY to 1.2**: Were exposure discontinuations or switches likely to be related to factors that are prognostic for the outcome?	/	
	**If N or PN to 1.3**, answer questions 1.4 to 1.6, which relate to baseline confounding		
	1.4. Did the authors use an appropriate analysis method that adjusted for all the critically important confounding areas?	NI	
	1.5. **If Y or PY to 1.4**: Were confounding areas that were adjusted for measured validly and reliably by the variables available in this study?	NI	
	1.6. Did the authors avoid adjusting for post-exposure variables?	NI	
	**If Y or PY to 1.3**, answer questions 1.7 and 1.8, which relate to time-varying confounding		

	1.7. Did the authors use an appropriate analysis method that adjusted for all the critically important confounding areas and for time-varying confounding?	/	
	1.8. **If Y or PY to 1.7**: Were confounding areas that were adjusted for measured validly and reliably by the variables available in this study?	/	
	**Risk of bias judgement**	Serious	Only scarce information is provided throughout the entire article
	Optional: What is the predicted direction of bias due to confounding?	Unpredictable	Some risk factors may be overestimated whereas other are underestimated and vice-versa
Bias in selection of participants into the study	2.1. Was selection of participants into the study (or into the analysis) based on variables measured after the start of the exposure?<br><br>**If N or PN to 2.1 go to 2.4**	N	
	2.2. **If Y/PY to 2.1:** Were the post-exposure variables that influenced selection associated with exposure?	/	
	2.3. **If Y/PY to 2.2:** Were the post-exposure variables that influenced eligibility selection influenced by the outcome or a cause of the outcome?	/	
	2.4 Do start of follow-up and start of exposure coincide for most participants?	N	
	2.5 If Y/PY to 2.2 and 2.3, or N/PN to 2.4: Were adjustment techniques used that are likely to correct for the presence of selection biases?	NI	
	**Risk of bias judgement**	Moderate	Only partial information is provided throughout the article
	Optional: What is the predicted direction of bias due to selection of participants into the study?	Favors experimental / Favors comparator / Towards null /Away from null / Unpredictable	[Rationale]

Bias in classification of exposures	3.1 Is exposure status well defined?	Y	
	3.2 Did entry into the study begin with start of the exposure?	N	
	3.3 Was information used to define exposure status recorded prior to outcome assessment?	NI	
	3.4 Could classification of exposure status have been affected by knowledge of the outcome or risk of the outcome?	PY	Could potentially have happened. No specific information throughout article
	3.5 Were exposure assessment methods robust (including methods used to input data)?	NI	No information provided on such assessment methods
	**Risk of bias judgement**	Moderate	Only partial information is provided throughout the article.
	Optional: What is the predicted direction of bias due to measurement of outcomes or exposures?	Favors experimental / Favors comparator / Towards null /Away from null / Unpredictable	[Rationale]
Bias due to departures from intended exposures	4.1. Is there concern that changes in exposure status occurred among participants?<br><br>**If your aim for this study is to assess the effect of initiating and adhering to an exposure (as in a per-protocol analysis), answer questions 4.2 and 4.3, otherwise continue to 4.4 if Y or PY to 4.1.**	N	
	4.2. Did many participants switch to other exposures?	N	
	4.3. Were the critical co-exposures balanced across exposure groups?	Y	
	4.4. **If NY/PN PY to 4.1, or Y/PY to 4.2, or 4.3:** Were adjustment techniques used that are likely to correct for these issues?	NI	
	**Risk of bias judgement**	Moderate	Few concerns present about questions 4.1 to 4.4

	Optional: What is the predicted direction of bias due to departures from the intended exposures?	Favors experimental / Favors comparator / Towards null /Away from null / Unpredictable	[Rationale]
Bias due to missing data	5.1 Were there missing outcome data?	NI	
	5.2 Were participants excluded due to missing data on exposure status?	NI	
	5.3 Were participants excluded due to missing data on other variables needed for the analysis?	NI	
	5.4 **<u>If Y/PY to 5.1, 5.2 or 5.3:</u>** Are the proportion of participants and reasons for missing data similar across exposures?	NI	
	5.5 **<u>If Y/PY to 5.1, 5.2 or 5.3:</u>** Were appropriate statistical methods used to account for missing data?	NI	
	**Risk of bias judgement**	Serious	A serious potential of bias is present as there is scarce information provided
	Optional: What is the predicted direction of bias due to missing data?	Favors experimental / Favors comparator / Towards null /Away from null / Unpredictable	[Rationale]
Bias in measurement of outcomes	6.1 Could the outcome measure have been influenced by knowledge of the exposure received?	Y	
	6.2 Was the outcome measure sensitive?	PN	Outcome variable subjectively assessed
	6.3 Were outcome assessors unaware of the exposure received by study participants?	N	
	6.4 Were the methods of outcome assessment comparable across exposure groups?	Y	
	6.5 Were any systematic errors in measurement of the outcome unrelated to exposure received?	NI	
	**Risk of bias judgement**	Moderate	Information only partly available throughout article.

	Optional: What is the predicted direction of bias due to measurement of outcomes?	Favors experimental / Favors comparator / Towards null /Away from null / Unpredictable	[Rationale]
Bias in selection of	Is the reported effect estimate likely to be selected, on the basis of the results, from...?		
the reported result	7.1. ... multiple outcome *measurements* within the outcome domain?	NI	[Description]
	7.2 ... multiple *analyses* of the exposure-outcome relationship?	NI	[Description]
	7.3 ... different *subgroups*?	NI	[Description]
	**Risk of bias judgement**	Serious	Potential serious risk since information is lacking throughout article
	Optional: What is the predicted direction of bias due to selection of the reported result?	Favors experimental / Favors comparator / Towards null /Away from null / Unpredictable	[Rationale]
Overall bias	**Risk of bias judgement**	Moderate / Serious	Bias could have entered at various stages of the work. Additionally, information is often scarce
	Optional:<br>What is the overall predicted direction of bias for this outcome?	Favors experimental / Favors comparator / Towards null /Away from null / Unpredictable	[Rationale]

Preliminary tool for risk of bias in exposure studies (2): For each study

Specify a target experiment specific to the study.

☐	The protocol-specified target experiment fully applies	*OR*	Participant	*1078 dairy cows*
			Experimental exposure	*Free stall housing exposed to a set of risk factors*
			Control exposure	*Free stall housing not exposed to a set of risk factors*

Specify the outcome

Specify which outcome is being assessed for risk of bias (typically from among those earmarked for the Summary of Findings table). Specify whether this is a proposed benefit or harm of exposure.

Risk factors of lameness in dairy cows (Possibly benefit and harm of exposure; probably more harm than benefit)

Is your aim for this study…?

- ☐ to assess the effect of initiating intervention (as in an intention-to-treat analysis)
- x to assess the effect of initiating and adhering to intervention (as in a per-protocol analysis)
- ☐ other (specify)

Specify the numerical result being assessed

In case of multiple alternative analyses being presented, specify the numeric result (e.g. RR = 1.52 (95% CI 0.83 to 2.77) and/or a reference (e.g. to a table, figure or paragraph) that uniquely defines the result being assessed.

Yaylak E, Akbas Y, Kaya I, Uzmay C. The fffects of several cow and herd level factors on lameness in Holstein cows reared in Izmir Province of Turkey. Journal Anim Vet Adv. 2010;9:2714-22. Table 3

Preliminary consideration of confounders

Complete a row for each important confounding area (i) listed in the review protocol; and (ii) relevant to the setting of this particular study, or which the study authors identified as potentially important.

"Important" confounding areas are those for which, in the context of this study, adjustment is expected to lead to a clinically important change in the estimated effect of the exposure. "Validity" refers to whether the confounding variable or variables fully measure the area, while "reliability" refers to the precision of the measurement (more measurement error means less reliability).

(xi) Confounding areas listed in the review protocol

Confounding area	Measured variable(s)	Is there evidence that controlling for this variable was unnecessary?*	Is the confounding area measured validly and reliably by this variable (or these variables)?	OPTIONAL: Is adjusting for this variable (alone) expected to move the effect estimate up or down?
Milk yield	Total milk yield	No	Yes	No information
	Milk yield per day	no		No information
Breed	Breed	No	Yes	No information
Stage of lactation	Days in milk	no		No information

(xii) **Additional confounding areas relevant to the setting of this particular study, or which the study authors identified as important**

Confounding area	Measured variable(s)	Is there evidence that controlling for this variable was unnecessary?*	Is the confounding area measured validly and reliably by this variable (or these variables)?	OPTIONAL: Is adjusting for this variable (alone) expected to move the effect estimate up or down?

No information provided in the study	No information provided in the study	No information provided in the study	No information provided in the study	No information provided in the study

* In the context of a particular study, variables can be demonstrated not to be confounders and so not included in the analysis: (a) if they are not predictive of the outcome; (b) if they are not predictive of exposure; or (c) because adjustment makes no or minimal difference to the estimated effect of the primary parameter. Note that "no statistically significant association" is not the same as "not predictive".

Preliminary consideration of criteria used to determine the accuracy of measurement of exposure and outcome

Complete a row for each measure listed in the study for the (i) exposure and (ii) outcome. Of the measures listed in the protocol, consider the sensitivity, specificity, and confidence in the methods used in the study.

(xi) Exposure measurement method listed in the study		
Method of measurement	Measured exposure	Is the exposure measured validly and reliably by this method (or these methods)?
Scoring	Body condition<br>Hygiene of lower legs	Partly yes (Subjective scoring system)
Computer records from Cattle Breeders' Association of Izmir	Parity, days in milk	No information
Interview with herd owner	Housing characteristics, feeding strategy, management facilities	No information

(xii) Outcome measurement method listed in the study		
Method of measurement	Measured outcome	Is the outcome measured validly and reliably by this method (or these methods)?
Scoring system	Locomotion/Lameness	Partly yes (Subjective scoring system)

Preliminary consideration of co-exposures

Complete a row for each important co-intervention (i) listed in the review protocol; and (ii) relevant to the setting of this particular study, or which the study authors identified as important.
"Important" co-interventions are those for which, in the context of this study, adjustment is expected to lead to a clinically important change in the estimated effect of the intervention.

**(xi) Co-exposures listed in the review protocol**		
Co-exposure	Is there evidence that controlling for this co-exposure was unnecessary (e.g., because it was not administered)?	Is presence of this co-exposure likely to favor outcomes in the experimental or the control group
Access to pasture	Yes	No information
Claw trimming	No	No information
Different housing conditions	Yes	No information

**(xii) Additional co-exposures relevant to the setting of this particular study, or which the study authors identified as important**		
Co-exposure	Is there evidence that controlling for this co-exposure was unnecessary (e.g., because it was not administered)?	Is presence of this co-exposure likely to favor outcomes in the experimental or the control group
No information	No information	No information

Risk of bias assessment (cohort-type studies)

Bias due to confounding	1.1 **Is there potential for confounding of the effect of exposure in this study? If N or PN to 1.1:** the study can be considered to be at low risk of bias due to confounding and no further signaling questions need be considered	Y	
	**If Y/PY to 1.1, answer 2.1 and 1.3 to determine whether there is a need to assess time-varying confounding:**		
	1.2. **If Y or PY to 1.1**: Was the analysis based on splitting follow up time according to exposure received?<br>**If N or PN to 1.2**, answer questions 1.4 to 1.6, which relate to baseline confounding	N	
	1.3. **If Y or PY to 1.2**: Were exposure discontinuations or switches likely to be related to factors that are prognostic for the outcome?	/	
	**If N or PN to 1.3**, answer questions 1.4 to 1.6, which relate to baseline confounding		
	1.4. Did the authors use an appropriate analysis method that adjusted for all the critically important confounding areas?	NI	
	1.5. **If Y or PY to 1.4**: Were confounding areas that were adjusted for measured validly and reliably by the variables available in this study?	NI	
	1.6. Did the authors avoid adjusting for post-exposure variables?	NI	
	**If Y or PY to 1.3**, answer questions 1.7 and 1.8, which relate to time-varying confounding		

	1.7. Did the authors use an appropriate analysis method that adjusted for all the critically important confounding areas and for time-varying confounding?	/	
	1.8. **If Y or PY to 1.7**: Were confounding areas that were adjusted for measured validly and reliably by the variables available in this study?	/	
	**Risk of bias judgement**	Serious	Only scarce information is provided throughout the entire article
	Optional: What is the predicted direction of bias due to confounding?	Unpredictable	Some risk factors may be overestimated whereas other are underestimated and vice-versa
Bias in selection of participants into the study	2.1. Was selection of participants into the study (or into the analysis) based on variables measured after the start of the exposure?<br><br>**If N or PN to 2.1 go to 2.4**	N	
	2.2. **If Y/PY to 2.1:** Were the post-exposure variables that influenced selection associated with exposure?	/	
	2.3. **If Y/PY to 2.2:** Were the post-exposure variables that influenced eligibility selection influenced by the outcome or a cause of the outcome?	/	
	2.4 Do start of follow-up and start of exposure coincide for most participants?	N	
	2.5 If Y/PY to 2.2 and 2.3, or N/PN to 2.4: Were adjustment techniques used that are likely to correct for the presence of selection biases?	NI	
	**Risk of bias judgement**	Moderate	Only partial information is provided throughout the article
	Optional: What is the predicted direction of bias due to selection of participants into the study?	Favors experimental / Favors comparator / Towards null /Away from null / Unpredictable	[Rationale]

Bias in classification of exposures	3.1 Is exposure status well defined?	Y	
	3.2 Did entry into the study begin with start of the exposure?	N	
	3.3 Was information used to define exposure status recorded prior to outcome assessment?	Y	
	3.4 Could classification of exposure status have been affected by knowledge of the outcome or risk of the outcome?	PY	Could potentially have happened. No specific information throughout article
	3.5 Were exposure assessment methods robust (including methods used to input data)?	NI	Not much information provided on such assessment methods. Implementation of subjective scoring systems.
	**Risk of bias judgement**	Moderate	Only partial information is provided throughout the article Also "PY" for 3.4
	Optional: What is the predicted direction of bias due to measurement of outcomes or exposures?	Favors experimental / Favors comparator / Towards null /Away from null / Unpredictable	[Rationale]
Bias due to departures from intended exposures	4.1. Is there concern that changes in exposure status occurred among participants?<br><br>**If your aim for this study is to assess the effect of initiating and adhering to an exposure (as in a per-protocol analysis), answer questions 4.2 and 4.3, otherwise continue to 4.4 if Y or PY to 4.1.**	N	
	4.2. Did many participants switch to other exposures?	NI	
	4.3. Were the critical co-exposures balanced across exposure groups?	NI	
	4.4. **If NY/PN PY to 4.1, or Y/PY to 4.2, or 4.3:** Were adjustment techniques used that are likely to correct for these issues?	NI	

	**Risk of bias judgement**	Serious	A serious potential of bias is present as there is scarce information provided
	Optional: What is the predicted direction of bias due to departures from the intended exposures?	Favors experimental / Favors comparator / Towards null /Away from null / Unpredictable	[Rationale]
Bias due to missing data	5.1 Were there missing outcome data?	NI	
	5.2 Were participants excluded due to missing data on exposure status?	NI	
	5.3 Were participants excluded due to missing data on other variables needed for the analysis?	NI	
	5.4 **If Y/PY to 5.1, 5.2 or 5.3:** Are the proportion of participants and reasons for missing data similar across exposures?	NI	
	5.5 **If Y/PY to 5.1, 5.2 or 5.3:** Were appropriate statistical methods used to account for missing data?	NI	
	**Risk of bias judgement**	Serious	A serious potential of bias is present as there is scarce information provided
	Optional: What is the predicted direction of bias due to missing data?	Favors experimental / Favors comparator / Towards null /Away from null / Unpredictable	[Rationale]
Bias in measurement of outcomes	6.1 Could the outcome measure have been influenced by knowledge of the exposure received?	Y	
	6.2 Was the outcome measure sensitive?	PN	Definition of the outcome variable was based on subjective scoring using a scoring system
	6.3 Were outcome assessors unaware of the exposure received by study participants?	PN	
	6.4 Were the methods of outcome assessment comparable across exposure groups?	Y	

	6.5 Were any systematic errors in measurement of the outcome unrelated to exposure received?	NI	
	**Risk of bias judgement**	Serious	Information only partly available throughout article.
	Optional: What is the predicted direction of bias due to measurement of outcomes?	Favors experimental / Favors comparator / Towards null /Away from null / Unpredictable	[Rationale]
Bias in selection of	Is the reported effect estimate likely to be selected, on the basis of the results, from...?		
the reported result	7.1. ... multiple outcome *measurements* within the outcome domain?	NI	[Description]
	7.2 ... multiple *analyses* of the exposure-outcome relationship?	NI	[Description]
	7.3 ... different *subgroups*?	NI	[Description]
	**Risk of bias judgement**	Serious	Potential serious risk since information is lacking throughout article
	Optional: What is the predicted direction of bias due to selection of the reported result?	Favors experimental / Favors comparator / Towards null /Away from null / Unpredictable	[Rationale]
Overall bias	**Risk of bias judgement**	Serious	In many parts, information on target questions is not available throughout the article
	Optional:<br>What is the overall predicted direction of bias for this outcome?	Favors experimental / Favors comparator / Towards null /Away from null / Unpredictable	[Rationale]
