# Supplementary material for: A systematic review and meta-analyses of risk factors associated with lameness in dairy cows
Source: BMC Vet Res. 2019 Oct 16;15:346. doi: 10.1186/s12917-019-2095-2 (PMC6796431; doi:10.1186/s12917-019-2095-2)
Supplement: Supplementary file 6 — Additional file 6. Funnel plots for the assessment of publication bias for each meta-analysis . [file 12917_2019_2095_MOESM6_ESM.pdf]

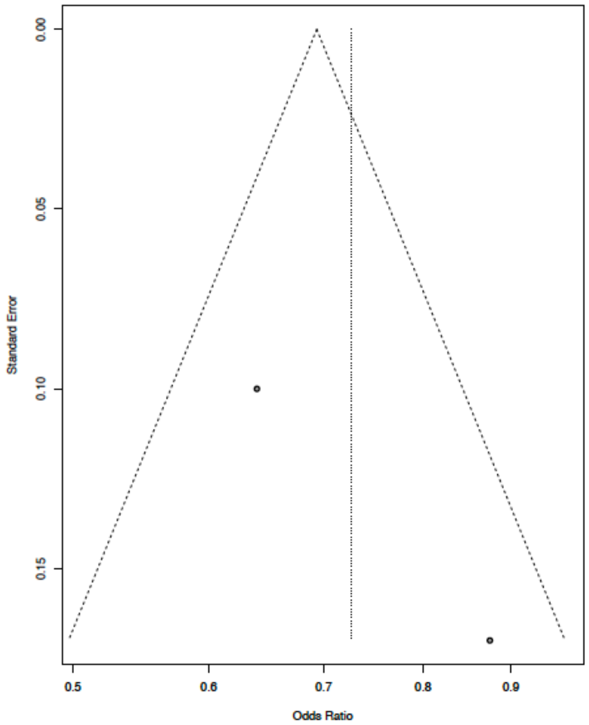

Funnel plot of the meta-analysis for BCS 3 vs BCS≤2.5

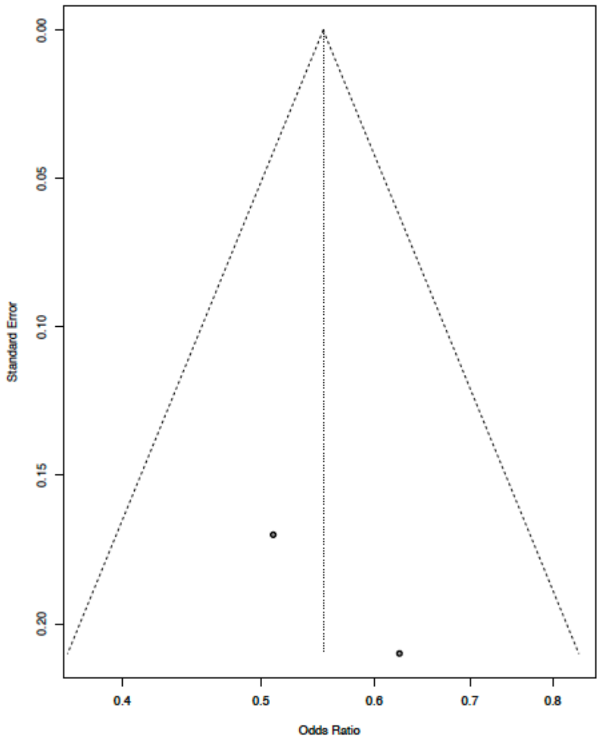

Funnel plot of the meta-analysis for BCS ≥3.5 vs BCS≤2.5

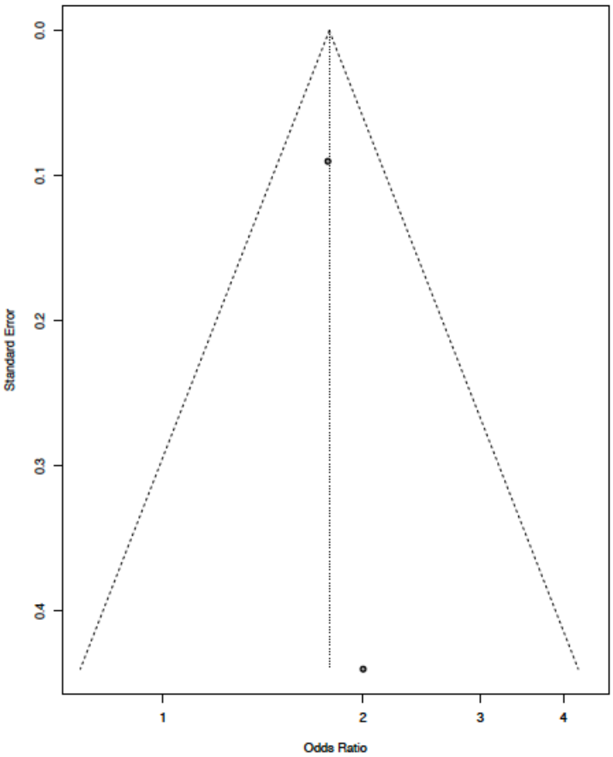

Funnel plot of the meta-analysis for the presence of claw overgrowth

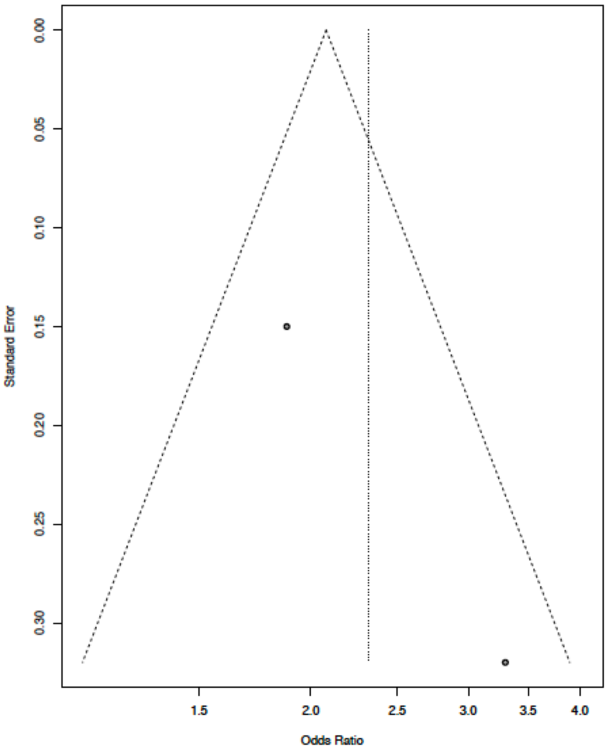

Funnel plot of the meta-analysis for stages of lactation (≤120 DIM vs. >120 DIM)

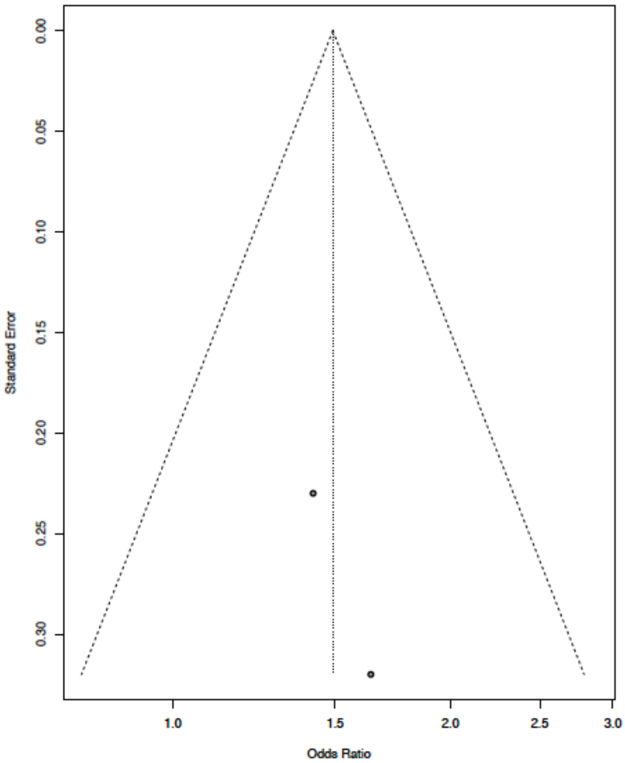

Funnel plot of the meta-analysis for herd size (30-50 animals vs. ≤29 animals)

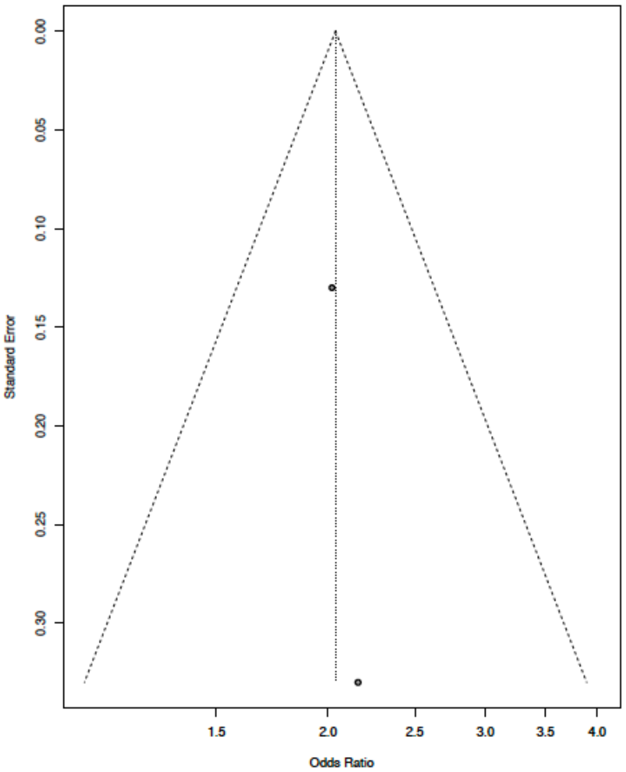

Funnel plot of the meta-analysis for herd size (≥50 animals vs. ≤29 animals)

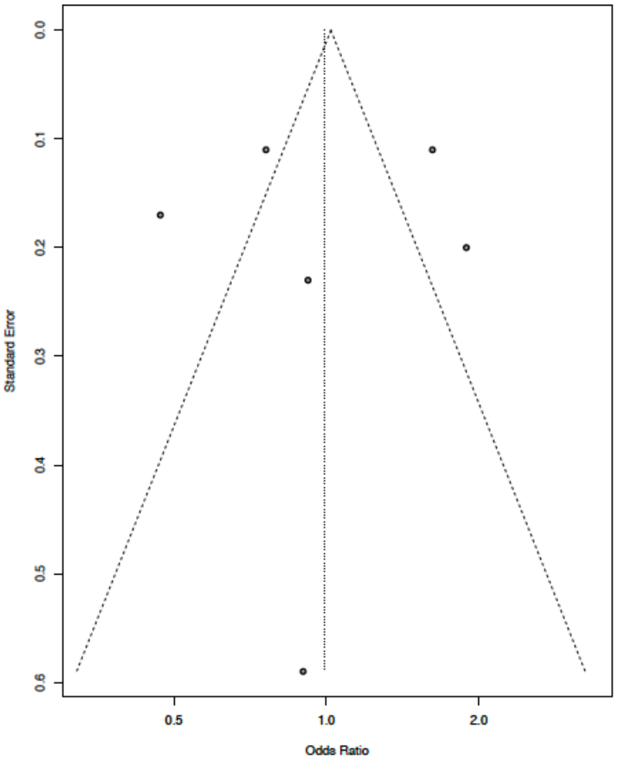

Funnel plot of the meta-analysis for parity (parity 2 vs. parity 1).

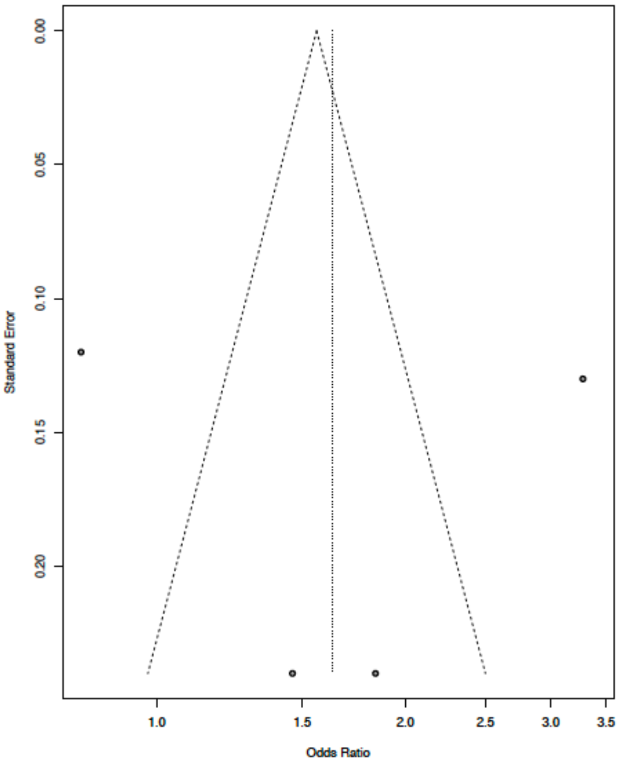

Funnel plot of the meta-analysis for parity (parity 3 vs. parity 1).

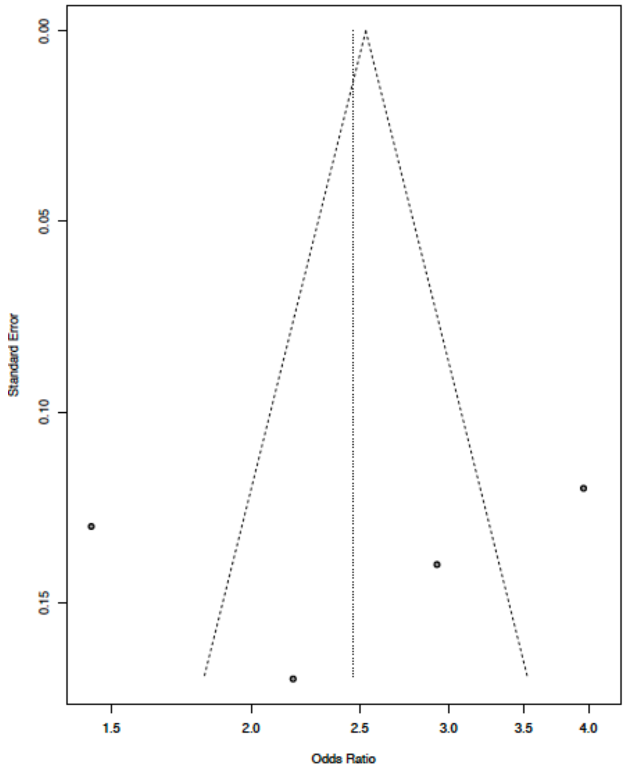

Funnel plot of the meta-analysis for parity (parity 4 vs. parity 1).
