# Supplementary material for: A systematic review and meta-analyses of risk factors associated with lameness in dairy cows
Source: BMC Vet Res. 2019 Oct 16;15:346. doi: 10.1186/s12917-019-2095-2 (PMC6796431; doi:10.1186/s12917-019-2095-2)
Supplement: Supplementary file 7 — Additional file 7. Pre-specified study protocol in compliance with the procedures presented by Shamseer et al. [78]. [file 12917_2019_2095_MOESM7_ESM.pdf]

PRISMA-P checklist 2015

Section 1: Administrative information
Title
Item 1a. Identification. Identify the report as a protocol of a systematic review.
Protocol for a systematic review and meta-analyses to identify risk factors associated with lameness in dairy cows housed in free stall barns and tie stall facilities.

Item 1b. Update. If the protocol is for an update of a previous systematic review, identify as such.
This is the first attempt for a systematic review in this context. No update of a previous systematic review was conducted.

Registration
Item 2. If registered, proved the name of registry (such as PROSPERO) and registration number.
Not applicable. PROSPERO is exclusive for studies in human patients according to the website. Even though the University of Nottingham has built a website for systematic reviews in the veterinary sector, an a priori registration was not possible on this website.

Authors
Item 3a. Contact information. Provide name, institutional affiliation, and email address of all protocol authors; provide physical mailing address of corresponding authors.
Corresponding author: Andreas W. Oehm
a.oehm@med.vetmed.uni-muenchen.de
Author Affiliations
1 Clinic for Ruminants with Ambulatory and Herd Health Services, Ludwig-Maximilians-Universität München, Sonnenstrasse 16, 85764 Oberschleissheim
2 Section of Epidemiology, Vetsuisse Faculty, University of Zurich, Winterthurerstrasse 270, CH 8057 Zurich
Email: Andreas W. Oehm a.oehm@med.vetmed.uni-muenchen.de
Sonja Hartnack sonja.hartnack@access.uzh.ch

Item 3b. Contributions. Describe contributions of protocol authors and identify the guarantor of the review.
AOE is the guarantor. AOE drafted the manuscript. AOE and SH contributed to the development of the selection criteria and the search strategy. SH and AR provided statistical expertise. All authors read, proved feedback, and approved the final manuscript.

Amendments
Item 4. If the report represents an amendment of a previously completed or published protocol, identify as such and indicate what changes were made; otherwise state plan for documenting important protocol amendments.
In the event of protocol amendments, the date of each amendment will be accompanied by a description of the change and the rationale.

Support
Item 5a. Sources. Indicate sources of financial or other support for the review.

There was no financial support of this study. The project was conducted in the context of a doctorate in accordance with the University of Munich. Salary was ensured to the doctoral student.

Item 5b: Sponsor. Proved the name of the review funder and/or sponsor.
Not applicable.

Item 5c Role of sponsor and/or funder. Describe roles of funder(s), sponsor(s), and/or institution(s), if any, in developing the protocol.
Not applicable.

Section 2: Introduction

Rationale

Item 6. Describe the rationale for the review in the context of what is already known.
Lameness in dairy cows remains a tremendous problem in modern dairy production all over no world regardless of the fact that an abundance of studies have been conducted on this issue. The confined artificial environment dairy cows are kept in is of crucial importance in the development of lameness problems within a herd. However due to the large number of studies evaluating risk factors of lameness in dairy cows, it is challenging to handle this deluge of information. Systematic reviews have become increasingly important in medicine since they provide the possibility of an organised compilation and appraisal of large bodies of evidence. Furthermore, some may be comparable and display sufficient information to statistically integrate their findings via meta-analysis. This helps to give definite answers to clinical questions and to summarise and present evidence. Although systematic reviews have been published in the context of dairy cow lameness, e.g. prevention and treatment of digital dermatitis, no systematic review has yet been conducted to collate information on risk factors associated with lameness in dairy cows. The European Food Safety Authority has presented an insightful report on the importance of the housing environment in the development of lameness problems in dairy cows. Therefore, the objective of the present work is to give a meticulous compilation and statistical evaluation of literature by means of a systematic review and meta-analysis on risk factors for lameness in dairy cows. We aim to contribute evidence to the current knowledge by giving an intricate exposition of literature as well as by providing a summary estimate of risk factor effects. Furthermore, areas of shortage of knowledge will be identified and outlined.

Objectives

Item 7. Provide an explicit statement of the question(s) the review will address with reference to participants, interventions, comparators, and outcome.
The aim of this systematic review is to identify risk factors associated with lameness in dairy cows that are housed in free stall barns and tie stall dairies.

Section 3: Methods

Eligibility criteria

Item 8. Specify the study characteristics (such as PICO, study design, setting, time frame) and report characteristics (such as years considered, language, publication status) to be used as criteria for eligibility for the review.
Published studies of all type will be selected if risk factors associated with lameness in dairy cows including alternative wording are described. We expect mainly observational studies to be abundant in this context. However, to retrieve as many potentially relevant articles as possible, no certain study type (e.g. clinical trials to evaluate the influence of certain floorings

or rubber mats) will be excluded. No time frame is imposed. Literature search will be performed from inception to February 27, 2018. Studies will be excluded if they are not available in Dutch, English, French, German, Italian, Portuguese or Spanish. Subsequently, studies will not be included if dairy cows are not housed in free stalls or tie stalls and if data cannot be retrieved sufficiently in order to conduct meta-analyses. Furthermore, studies need to meet the reporting characteristics suggested by the STROBE guidelines to enter this review.

Information source

Item 9. describe all intended information sources (such as electronic databases, contact with study authors, trial registers, or other grey literature sources) with planned dates of coverage.

Literature searches will be conducted for five electronic data bases (MEDLINE (incl. Epub ahead of print, In process and other non-indexed citations), Web of Science, BIOSIS Previews, AGRICOLA, VETMED RESOURCE/CABI (https://www.cabi.org/VetMedResource/).

Search strategy

Item 10. Present draft of search strategy to be used for at least one electronic database, including planned limits such that it could be repeated.

Both qualitative and quantitative studies will be sought. No study designs or language limits will be imposed on the search, although only studies available in Dutch, English, French, German, Italian, Portuguese or Spanish will be included due to resource limits. (MEDLINE (incl. Epub ahead of print, In process and other non-indexed citations), Web of Science, BIOSIS Previews, AGRICOLA, VETMED RESOURCE/CABI (https://www.cabi.org/VetMedResource/) will be searched. The specific search strategy will be created by a professional health sciences librarian with expertise in systematic review searching. The search strategy will be developed with input from AOE and SH. The search terms listed below will be implemented:

1. ("dairy cow" OR "dairy cows" OR "dairy farm" OR "dairy farms" OR "dairy herd" OR "dairy herds" OR "dairy cattle") AND
2. (lame* OR ((impaired OR alter* OR disturb*) AND
3. (gait OR locomotion))) AND
4. (((risk OR management OR "herd-level") AND factor*) OR prevalence OR associat*)

Study records

Item 11a. Data management. Describe the mechanism(s) that will be used to manage records and data throughout the review.

Not applicable.

Item 11b. Selection process. State the process that will be used for selecting studies (such as two independent reviewers) through each phase of the review (Screening, eligibility, and inclusion in meta-analysis).

Two authors (AOE, AS) will independently screen titles and abstracts of primary articles yielded by the search against the inclusion criteria. In case of disagreement, GKS will decide upon inclusion. Full texts will be obtained for all articles that appear to be available and in

compliance with the inclusion criteria. The full texts will then be screened by AOE (and the decision checked by AS) whether these meet the inclusion criteria. Reporting quality will be assessed using the STROBE checklists. Additional information will be attempted to be obtained from primary study authors where necessary. Neither of the review authors will be blind to the journal titles or to the study authors or institutions.

Item 11c. Data collection process. Describe planned method of extracting data from reports (such as piloting forms, done independently, in duplicate), any processes for obtaining and confirming data from investigators.
Data extraction will be performed by AOE after initial discussion with SH and AR. Data extraction will include information on author and publication date, country, risk factors for lameness in dairy cows, definition of lameness and applied locomotion scoring system, number of animals, housing system and funding of the research project. Data will be extracted and collected using using Microsoft Excel 2016 (macOS) containing information on author(s), study title, year of publication, country, group sizes i.e. absolute number or percentage of lame and sound animals with regard to different risk factors, confidence intervals, standard errors of odds ratios, and coefficients, odds ratios and p-values. Studies authors will be contacted to access data if necessary.

Data items
Item 12. List and define all variables for which data will be sought (such as PICO items, funding sources) and any pre-planned data assumptions and simplifications.
Data extraction will include information on author and publication date, country, risk factors for lameness in dairy cows, definition of lameness and applied locomotion scoring system, number of animals, housing system and funding of the research project. The latter information will be retrieved according to AMSTAR and because potentially clinical trials evaluating interventions may receive funding from the industry. Data will be extracted and collected using using Microsoft Excel 2016 (macOS) containing information on author(s), study title, year of publication, country, group sizes i.e. absolute number or percentage of lame and sound animals with regard to different risk factors, confidence intervals, standard errors of odds ratios, and coefficients, odds ratios and p-values. Studies authors will be contacted to access data if necessary. If necessary, calculations from available data will be made in order to obtain values necessary for meta-analyses.

Outcomes and prioritisation
Item 13. List and define all outcomes of which data will be sought, including prioritization of main and additional outcomes, with rationale.
The primary outcome will be dairy cows that are lame due to the occurrence of certain risk factors. However, we expect, that the definition of lameness will be very different across studies and hence be a problem Alternative wording is permitted, since we expect a large variety of nomenclature. We plan not to focus on one single definition of lameness in order to not exclude potentially relevant articles in regard to this definition.

Risk of bias individual studies
Item 14. Describe anticipated methods for assessing risk of bias of individual studies, including whether this will be done at the outcome or study level, or both; state how this information will be used in data synthesis.
Graphic representation of potential bias across studies will be computed creating funnel plots via the statistical software 'R'.

Data synthesis

Item 15a. Describe criteria under which study data will be quantitatively synthesized.
If studies are sufficiently homogeneous in terms of design and comparator and if they provide enough statistical information to be used for quantitative synthesis, meta analyses will be performed using random-effects models.

Item 15b. If data are appropriate for synthesis, describe planned summary measures, methods of handling data, and methods of combining data from studies, including any planned exploration of consistency (such as I^2, Kendall's τ^2)
Odds ratios (OR) and their 95% confidence intervals will be calculated. Heterogeneity will be displayed in the forest plots by I^2 and τ^2).

Item 15c: Describe any proposed additional analyses (e.g. sensitivity or subgroup analyses, meta-regression).
Subgroup analyses or other additional analyses are not planned.

Meta-bias(es)
Item 16. Specify any planned assessment of meta-bias(es) (such as publication bias across studies, selective reporting within studies.
Graphic representation of potential bias across studies will be computed creating funnel plots via the statistical software 'R'.

Confidence and cumulative estimate
Item 17. Describe how the strength of the body of evidence will be assessed (such as GRADE).
Reporting quality will be assessed using the STROBE guidelines.
