# Supplementary material for: A systematic review and meta-analyses of risk factors associated with lameness in dairy cows
Source: BMC Vet Res. 2019 Oct 16;15:346. doi: 10.1186/s12917-019-2095-2 (PMC6796431; doi:10.1186/s12917-019-2095-2)
Supplement: Supplementary file 8 — Additional file 8. Calculation of standard errors for the meta-analysis of BCS. [file 12917_2019_2095_MOESM8_ESM.pdf]

The standard error was calculated as the square root from values obtained applying the following procedure

$$\sqrt{\frac{1}{n_{\text{sound BCS 3.0}}}+\frac{1}{n_{\text{lame BCS 3.0}}}+\frac{1}{n_{\text{sound BCS 2.5}}}+\frac{1}{n_{\text{lame BCS 2.5}}}}$$

and

$$\sqrt{\frac{1}{n_{\text{sound BCS 3.5}}}+\frac{1}{n_{\text{lame BCS 3.5}}}+\frac{1}{n_{\text{sound BCS 2.5}}}+\frac{1}{n_{\text{lame BCS 2.5}}}}$$
