# Supplementary material for: A systematic review and meta-analyses of risk factors associated with lameness in dairy cows
Source: BMC Vet Res. 2019 Oct 16;15:346. doi: 10.1186/s12917-019-2095-2 (PMC6796431; doi:10.1186/s12917-019-2095-2)
Supplement: Supplementary file 11 — Additional file 11. Reference list of studies listed in Additional files 2, 3, and 4. [file 12917_2019_2095_MOESM11_ESM.pdf]

1. Adams AE, Lombard JE, Fossler CP, Roman-Muniz IN, Kopral CA. Associations between housing and management practices and the prevalence of lameness, hock lesions, and thin cows on US dairy operations. J Dairy Sci. 2017;100:2119-36.
2. Alban L. Lameness in Danish dairy cows - Frequency and possible risk factors. Prev Vet Med. 1995;22:213-25.
3. Alban L, Agger JF, Lawson LG. Lameness in tied Danish dairy cattle: The possible influence of housing systems, management, milk yield, and prior incidents of lameness. Preve Vet Med. 1996;29:135-49.
4. Alcañiz A. Zinc glycinate supplementation and incidence of lameness in dairy cows. Albéitar. 2014:68-9.
5. Andreasen SN, Forkman B. The welfare of dairy cows is improved in relation to cleanliness and integument alterations on the hocks and lameness when sand is used as stall surface. J Dairy Sci. 2012;95:4961-7.
6. Baird LG, O'Connell NE, McCoy MA, Keady TWJ, Kilpatrick DJ. Effects of breed and production system on lameness parameters in dairy cattle. J Dairy Sci. 2009;92:2174-82.
7. Battagin M, Sartori C, Biffani S, Penasa M, Cassandro M. Genetic parameters for body condition score, locomotion, angularity, and production traits in Italian Holstein cattle. J Dairy Sci. 2013;96:5344-51.
8. Becker J, Steiner A, Kohler S, Koller-Bahler A, Wuthrich M, Reist M. Lameness and foot lesions in Swiss dairy cows: I. Prevalence. Schweiz Arch Tierheilkd. 2014a;156:71-8.
9. Becker J, Steiner A, Kohler S, Koller-Bahler A, Wuthrich M, Reist M. Lameness and foot lesions in Swiss dairy cows: II. Risk factors. Schweiz Arch Tierheilkd. 2014b;156:79-89.
10. Bergsten C. Effects of conformation and management system on hoof and leg diseases and lameness in dairy cows. Vet Clin North Am-Food Anim Pract. 2001;17:1-23.
11. Bergsten C. Lameness and claw lesions as influenced by stall environment and cow comfort. Médecin Vét Québec. 2004;34:29-31.
12. Bergsten C. Locomotion, preference and hygiene on alternative flooring surfaces - research and experiences from EU Lame Cow and other studies. 2010. Cattle Lameness Conference, Nottingham, UK: 4-11
13. Bernardi F, Fregonesi J, Winckler C, Veira DM, von Keyserlingk MAG, Weary DM. The stall-design paradox: Neck rails increase lameness but improve udder and stall hygiene. J Dairy Sci. 2009;92:3074-80.
14. Bhadauria P, Lathwal SS, Jadoun YS, Ruhil AP, Devi I, Gupta R. Effect of transition diet fortification on lameness and blood metabolites in pre- and post- partum lame Karan Fries cows. Indian J Anim Scis. 2015;85:1006-11.
15. Bicalho, R. C., S. H. Cheong, G. Cramer, and C. L. Guard. Association between a visual and an automated locomotion score in lactating Holstein cows. J. Dairy Sci. 2007;90:3294-3300.
16. Bielfeldt JC, Badertscher R, Tolle KH, Krieter J. Risk factors influencing lameness and claw disorders in dairy cows. Livest Prod Sci. 2005;95:265-71.
17. Blowey RW. The effects of season and stage of lactation on lameness in 900 dairy cows. Proceedings of the 13th International Symposium and 5th Conference on Lameness in Ruminants, Maribor, Slovenija, 11th-15th February 2004:43-5.
18. Blowey R. Factors associated with lameness in dairy cattle. In Pract. 2005;27:154-62.
19. Boelling D. The genetics of feet, legs and locomotion in cattle. Anim Breed Abstr. 1997;65:1-11.
20. Boettcher PJ, Dekkers JCM, Warnick LD, Wells SJ. Genetic analysis of clinical lameness in dairy cattle. J Dairy Sci. 1998;81:1148-56.

21. Bouffard V, de Passille AM, Rushen J, Vasseur E, Nash CGR, Haley DB, et al. Effect of following recommendations for tiestall configuration on neck and leg lesions, lameness, cleanliness, and lying time in dairy cows. J Dairy Sci. 2017;100:2935-43.
22. Bramley E, Costa ND, Fulkerson WJ, Lean IL. Associations between body condition, rumen fill, diarrhoea and lameness and ruminal acidosis in Australian dairy herds. New Zealand Vet J. 2013;61:323-9.
23. Brinkmann J. Influence of the housing system on lameness prevalence in organic dairy farming. Proceedings of the 13th International Symposium and 5th Conference on Lameness in Ruminants, Maribor, Slovenija, 11th-15th February 2004: 166-7.
24. Burgstaller J, Raith J, Kuchling S, Mandl V, Hund A, Kofler J. Claw health and prevalence of lameness in cows from compost bedded and cubicle freestall dairy barns in Austria. Vet J. 2016;216:81-6.
25. Candiani D. EFSA's scientific opinion on factors affecting leg and locomotion disorders in dairy cows Sustainable animal husbandry: prevention is better than cure, Volume 1. Proceedings of the 14th International Congress of the International Society for Animal Hygiene (ISAH), Vechta, Germany, 19th to 23rd July 2009: 383-6.
26. Chanel NM. The effects of flunixin meglumine treatment and hoof trimming on lying behavior and locomotion in dairy cows. Proceedings of the 47th Annual Conference of the American Association of Bovine Practitioners, Albuquerque, New Mexico, USA, 18th-20th September 2014: 133-4.
27. Chapinal, N., A. M. de Passille, D. M. Weary, M. A. von Keyserlingk, and J. Rushen. 2009. Using gait score, walking speed, and lying behavior to detect hoof lesions in dairy cows. J.Dairy Sci. 92(9):4365-4374.
28. Chapinal N, Barrientos AK, von Keyserlingk MAG, Galo E, Weary DM. Herd-level risk factors for lameness in freestall farms in the northeastern United States and California. J Dairy Sci. 2013;96:318-28.
29. Chapinal N, von Keyserlingk MAG, Cerri RLA, Ito K, LeBlanc SJ, Weary DM. Short communication: Herd-level reproductive performance and its relationship with lameness and leg injuries in freestall dairy herds in the northeastern United States. J Dairy Sci. 2013;96:7066-72.
30. Chapinal N, Liang Y, Weary DM, Wang Y, von Keyserlingk MAG. Risk factors for lameness and hock injuries in Holstein herds in China. J Dairy Sci. 2014;97:4309-16.
31. Cook NB. Prevalence of lameness among dairy cattle in Wisconsin as a function of housing type and stall surface. J Am Vet Med Assoc. 2003;223:1324-8.
32. Cook NB, Bennett TB, Nordlund KV. Effect of free stall surface on daily activity patterns in dairy cows with relevance to lameness prevalence. J Dairy Sci. 2004a;87:2912-22.
33. Cook NB, Bennett TB, Nordlund, KV. Using indices of cow comfort to predict stall use and lameness. Proceedings of 13th International Ruminant Lameness Symposium, Maribor, Slovenia 11th-15th February 2004b: 162-164.
34. Cook NB, Hess JP, Foy MR, Bennett TB, Brotzman RL. Management characteristics, lameness, and body injuries of dairy cattle housed in high-performance dairy herds in Wisconsin. J Dairy Sci. 2016;99:5879-91.
35. Costa JHC, Burnett TA, von Keyserlingk MAG, Hotzel MJ. Prevalence of lameness and leg lesions of lactating dairy cows housed in southern Brazil: Effects of housing systems. J Dairy Sci. 2018;101:2395-405.
36. DairyCo, DairyCo Mobility Score. 2007. DairyCo , Kenilworth
37. Dembele I, Spinka M, Stehulova I, Panama J, Firla P. Factors contributing to the incidence and prevalence of lameness on Czech dairy farms. Czech J Anim Sci. 2006;51:102-9.

38. Demirkan I. Skin diseases of the bovine digit associated with lameness. Vet Bull. 2000;70:149-71.
39. Dippel S, Dolezal M, Brenninkmeyer C, Brinkmann J, March S, Knierim U, et al. Risk factors for lameness in freestall-housed dairy cows across two breeds, farming systems, and countries. J Dairy Sci. 2009a;92:5476-86.
40. Dippel S, Dolezal M, Brenninkmeyer C, Brinkmann J, March S, Knierim U, et al. Risk factors for lameness in cubicle housed Austrian Simmental dairy cows. Prev Vet Med. 2009b;90:102-12.
41. Doherty N, More SJ, Somers J. Short communication: Risk factors for lameness on 10 dairy farms in Ireland. Vet Rec. 2014;174.
42. Eckelkamp EA, Taraba JL, Akers KA, Harmon RJ, Bewley JM. Sand bedded freestall and compost bedded pack effects on cow hygiene, locomotion, and mastitis indicators. Livest Sci. 2016;190:48-57.
43. Endres MI. The relationship of cow comfort and flooring to lameness disorders in dairy cattle. Vet Clin North Am-Food Anim Pract. 2017;33:227-33.
44. Espejo LA, Endres MI, Salfer JA. Prevalence of lameness in high-producing Holstein cows housed in freestall barns in Minnesota. J Dairy Sci. 2006;89:3052-8.
45. Espejo LA, Endres MI. Herd-level risk factors for lameness in high-producing Holstein cows housed in freestall barns. J Dairy Sci. 2007;90:306-14.
46. Faye B. Environmental factors associated with lameness and limb disease in French dairy herds. Environment and animal health. Volume 1. Proceedings of the 6th International Congress on Animal Hygiene, 14th-17th June 1988, Skara, Sweden. 65-75.
47. Faye B, Lescourret F. Environmental factors associated with lameness in dairy cattle. Prev Vet Med. 1989;7:267-87.
48. Fjeldaas T, Sogstad AM, Osteras O. Claw trimming routines in relation to claw lesions, claw shape and lameness in Norwegian dairy herds housed in tie stalls and free stalls. Prev Vet Med. 2006;73:255-71.
49. Flower, F. C. and D. M. Weary. Effect of hoof pathologies on subjective assessments of dairy cow gait. J. Dairy Sci. 2006a;89:139-146.
50. Flower F, Sanderson D, Weary D. Effects of milking on dairy cow gait. J Dairy Sci. 2006b;89:2084-9.
51. Foditsch C, Oikonomou G, Machado VS, Bicalho ML, Ganda EK, Lima SF, et al. Lameness prevalence and risk factors in large dairy farms in Upstate New York. Model development for the prediction of claw horn disruption lesions. PLoS One. 2016;11(1):e0146718.
52. Frankena K, Somers J, Schouten WGP, van Stek JV, Metz JHM, Stassen EN, Graat EAM. The effect of digital lesions and floor type on locomotion score in Dutch dairy cows. Prev Vet Med. 2009;88:150-7.
53. Gibbons, J., D. B. Haley, J. H. Cutler, C. Nash, J. Z. Heyerhoff, D. Pellerin, S. Adam, A. Fournier, A. M. de Passillé, and J. Rushen. A comparison of 2 methods of assessing lameness prevalence in tiestall herds. J. Dairy Sci. 2014;97:350-353.
54. Green LE, Borkert J, Monti G, Tadich N. Associations between lesion-specific lameness and the milk yield of 1,635 dairy cows from seven herds in the Xth region of Chile and implications for management of lame dairy cows worldwide. Anim Welfare. 2010;19:419-27.
55. Green LE, Huxley JN, Banks C, Green MJ. Temporal associations between low body condition, lameness and milk yield in a UK dairy herd. Prev Vet Med. 2014;113:63-71.
56. Groehn JA, Kaneene JB, Foster D. Risk factors associated with lameness in lactating dairy cattle in Michigan. Prev Vet Med. 1992;14:77-85.

57. Gudaj R. Associations between the occurrence of lameness, number of orthopaedic blocks used by hoof trimmers and management risk factors in dairy cow herds. Anim Welfare, Etológia és tartástechnológia. 2012;8:223-39.
58. Hedges J, Blowey RW, Packington AJ, Ocallaghan CJ, Green LE. A longitudinal field trial of the effect of biotin on lameness in dairy cows. J Dairy Sci. 2001;84:1969-75.
59. Hedges VJ. Biotin: an overview of recent research into its effect on lameness in the dairy cow. Cattle Pract. 2002;10:157-61.
60. Hernandez-Mendo O, von Keyserlingk MAG, Veira DM, Weary DM. Effects of pasture on lameness in dairy cows. J Dairy Sci. 2007;90:1209-14.
61. Hettich E, Hinostroza MF, van Schaik G, Tadich N. Factors associated to lameness in 50 dairy herds in the X-th Region, Chile. Arch Med Vet. 2007;39:247-53.
62. Hirst WM, Murray RD, Ward WR, French NP. A mixed-effects time-to-event analysis of the relationship between first-lactation lameness and subsequent lameness in dairy cows in the UK. Prev Vet Med. 2002;54:191-201.
63. Hultgren J. Lameness and udder health in Swedish dairy herds, as influenced by housing changes. Acta Vet Scand. 2003;44:P61.
64. Hultgren, J., T. Manske, C. Bergsten. Associations of sole ulcer at claw trimming with reproductive performance udder health, and culling in Swedish dairy cattle. Prev Vet Med. 2004;62:233-251
65. Hultgren J. Alley-floor design, claw lesions and locomotion in Swedish loose-housed dairy cattle. Volume 1. Proceedings of the 13th International Congress in Animal Hygiene, Tartu, Estonia, 17th-21st June, 2007: 58-64 .
66. Huxley JN. Lameness in dairy cows: impact of practical nutritional and environmental management. Recent Advances in Animal Nutrition. 41st University of Nottingham Feed Conference, Nottingham, UK, 4th-6th September 2007: 75-89.
67. Kilic N, Ceylan A, Serin I, Gokbulut C. Possible interaction between lameness, fertility, some minerals, and vitamin E in dairy cows. Bull Vet Inst Pulawy. 2007;51:425-9.
68. King MTM, LeBlanc SJ, Pajor EA, DeVries TJ. Cow-level associations of lameness, behavior, and milk yield of cows milked in automated systems. J Dairy Sci. 2017;100:4818-28.
69. King MTM, Pajor EA, LeBlanc SJ, DeVries TJ. Associations of herd-level housing, management, and lameness prevalence with productivity and cow behavior in herds with automated milking systems. J Dairy Sci. 2016;99:9069-79.
70. Köck A, Fuerst-Waltl B, Steininger F, Egger-Danner C. Genetic parameters for body weight, body condition score and lameness in Austrian dairy cows. Interbull Bull. 2016(50).
71. Leach, K. A., S. Dippel, J. Huber, S. March, C. Winckler, and H. R. Whay. Assessing lameness in cows kept in tie-stalls. J. Dairy Sci. 2009;92:1567-1574.
72. Lean IJ, Westwood CT, Golder HM, Vermunt JJ. Impact of nutrition on lameness and claw health in cattle. Livest Sci. 2013;156:71-87.
73. Lim PY, Huxley JN, Willshire JA, Green MJ, Othman AR, Kaler J. Unravelling the temporal association between lameness and body condition score in dairy cattle using a multistate modelling approach. Prev Vet Med. 2015;118:370-7.
74. Logue D. The impact of nutrition on lameness - a review. 2011 Cattle Lameness Conference, Loughborough, UK, 13th April 2011: 1-11.
75. Logue DN. A preliminary review of the effects of environment and behaviour on lameness in the dairy cow. Proceedings of the 12th International Symposium on Lameness in Ruminants, Orlando, Florida, USA, 9th-13th January 2002: 18-26.

76. Mahendran S, editor The effect of pre-calving trims on subsequent lameness episodes in dairy heifers. 2015 Cattle Lameness Conference, 22 April 2015, Warriors Way, Worcester, UK; 2015: 17-22.
77. Manske T. Hoof lesions and lameness in Swedish dairy cattle : prevalence, risk factors, effects of claw trimming, and consequences for productivity. PhD thesis. Acta Universitatis Agriculturae Sueciae 135: Skara : Swedish University of Agricultural Sciences, 2002.
78. Manske, T., J. Hultgren, and C. Bergsten. Prevalence and interrelationships of hoof lesions and lameness in Swedish dairy cows. Prev Vet Med. 2002a;54:247-263
78. Manske, T., J. Hultgren, and C. Bergsten. A cross-sectional study of risk factors for the hoof health of Swedish dairy cattle. 2002b;Unpublished.
80. Manske, T., J. Hultgren, and C. Bergsten. The effect of claw trimming on the hoof health of Swedish dairy cattle. Prev Vet Med. 2002c;54:113-129.
81. Manson, F. a. and J. Leaver. The influence of concentrate amount on locomotion and clinical lameness in dairy cattle. Anim Sci. 1988;47:185-190.
82. Morabito E, Barkema HW, Pajor EA, Solano L, Pellerin D, Orsel K. Effects of changing freestall area on lameness, lying time, and leg injuries on dairy farms in Alberta, Canada. J Dairy Sci. 2017;100:6516-26.
83. Mudroň P. Role of ketosis in lame dairy cows. XIII Middle European Buiatrics Congress, Belgrade, Serbia, 5th-8th June, 2013: 90-7.
84. Mülling CKW. Risk factors associated with foot lameness in dairy cattle and a suggested approach for lameness reduction. 24th World Buiatrics Congress, Nice, France, 15th-19th October, 2006:118-29.
85. Murray R. Researching risk factors associated with cattle lameness. Vet J. 2000;159:109-10.
86. Newsome RF, Green MJ, Bell NJ, Bollard NJ, Mason CS, Whay HR, et al. A prospective cohort study of digital cushion and corium thickness. Part 2: Does thinning of the digital cushion and corium lead to lameness and claw horn disruption lesions? J Dairy Sci. 2017;100:4759-71.
87. O'Callaghan, K., P. Cripps, D. Downham, and R. Murray. Subjective and objective assessment of pain and discomfort due to lameness in dairy cattle. Anim Welfare 2003;12:605-610.
88. O'Driscoll KKM, Hanlon A, French P, Boyle LA. The effects of two out-wintering pad systems compared with free-stalls on dairy cow hoof and limb health. J Dairy R. 2009;76:59-65.
89. O'Driscoll KKM, Schutz MM, Lossie AC, Eicher SD. The effect of floor surface on dairy cow immune function and locomotion score. J Dairy Sci. 2009;92:4249-61.
90. Offer JE. A comparison of two concentrate formulations on lameness and hoof health in first and second lactation dairy cows Proceedings of the 12th International Symposium on Lameness in Ruminants, Orlando, Florida, USA, 9th-13th January 2002:259-62 .
91. Offer JE, Logue DN, Offer NW, Marsden M. The effect of concentrate composition on lameness and hoof health in dairy cows. Vet J. 2004;167:111-3.
92. Offer JE, Logue DN, Roberts DJ. The effect of protein source on lameness and solear lesion formation in dairy cattle. Anim Sci. 1997;65:143-9.
93. Oikonomou G, Michailidis G, Kougioumtzis A, Avdi M, Banos G. Effect of polymorphisms at the STAT5A and FGF2 gene loci on reproduction, milk yield and lameness of Holstein cows. Research in Vet Sci. 2011;91:235-9.
94. Olechnowicz J, Jaskowski JM. Body condition related to lameness in dairy cows. Medycyna Weterynaryjna. 2014;70:353-6.

95. Olechnowicz J, Jaskowski JM. Risk factors influencing lameness and key areas in reduction of lameness in dairy cows. Medycyna Weterynaryjna. 2010;66:507-11.
96. Onyiro OM, Brotherstone S. Genetic analysis of locomotion and associated conformation traits of Holstein-Friesian dairy cows managed in different housing systems. J Dairy Sci. 2008;91:322-8.
97. Onyiro OM, Offer J, Brotherstone S. Risk factors and milk yield losses associated with lameness in Holstein-Friesian dairy cattle. Animal. 2008;2:1230-7.
98. Penev T, Mitev J, Iliev A, Borisov I, Miteva T, Gergovska Z, et al. Hygienic and technological conditions favouring lameness in dairy cows: a review. Rev Med Vet. 2012;163:499-504.
99. Perez-Cabal MA, Alenda R. Clinical lameness and risk factors in a Spanish Holstein population. Livest Sci. 2014;164:168-74.
100. Philipot JM, Pluvinage P, Cimarosti I, Sulpice P, Bugnard F. Risk factors of dairy cow lameness associated with housing conditions. Vet Res. 1994;25:244-8.
101. Popescu S. Exercise effect on lameness prevalence in tied dairy cows
Animal hygiene and sustainable livestock production. Volume 3. Proceedings of the XVth International Congress of the International Society for Animal Hygiene, Vienna, Austria, 3th-7th July 2011:1089-91.
102. Popescu S. Relationship between barn hygiene and lameness prevalence in thirty-five Transylvanian dairy farms. Bulletin of University of Agricultural Sciences and Veterinary Medicine Cluj-Napoca. 2010;67:222-8.
103. Potzsch CJ, Collis VJ, Blowey RW, Packington AJ, Green LE. The impact of parity and duration of biotin supplementation on white line disease lameness in dairy cattle. J Dairy Sci. 2003;86:2577-82.
104. Randall LV, Green MJ, Green LE, Chagunda MGG, Mason C, Archer SC, et al. The contribution of previous lameness events and body condition score to the occurrence of lameness in dairy herds: A study of 2 herds. J Dairy Sci. 2018;101:1311-24.
105. Richert RM, Cicconi KM, Gamroth MJ, Schukken YH, Stiglbauer KE, Ruegg PL. Perceptions and risk factors for lameness on organic and small conventional dairy farms. J Dairy Sci. 2013;96:5018-26.
106. Ristevski M, Toholj B, Cincovic M, Bobos S, Trojacanec P, Stevancevic M, Ozren S. Influence of body condition score and ultrasound-determined thickness of body fat deposit in Holstein-Friesian cows on the risk of lameness developing. Kafkas Universitesi Veteriner Fakultesi Dergisi. 2017a;23:69-75.
107. Ristevski M, Toholj B, Cincovic M, Trojacanec P, Staric J, Smolec O. Milk production, body condition score and metabolic parameters at the peak of lactation as risk factors for chronic lameness in dairy cows. Kafkas Universitesi Veteriner Fakultesi Dergisi. 2017b;23:721-7.
108. Rouha-Mulleder C, Iben C, Wagner E, Laaha G, Troxler J, Waiblinger S. Relative importance of factors influencing the prevalence of lameness in Austrian cubicle loose-housed dairy cows. Prev Vet Med. 2009;92:123-33.
109. Rowlands G, Russell A, Williams L. Effects of season, herd size, management system and veterinary practice on the lameness incidence in dairy cattle. Vet Rec. 1983;113:441-5.
110. Sadiq MB, Ramanoon SZ, Mansor R, Syed-Hussain SS, Mossadeq WMS. Prevalence of lameness, claw lesions, and associated risk factors in dairy farms in Selangor, Malaysia. Trop Anim Health Prod. 2017;49:1741-8.
111. Sanders AH, Shearer JK, De Vries A. Seasonal incidence of lameness and risk factors associated with thin soles, white line disease, ulcers, and sole punctures in dairy cattle. J Dairy Sci. 2009;92:3165-74.

112. Sarjokari K, Kaustell KO, Hurme T, Kivinen T, Peltoniemi OAT, Saloniemi H, Rajala-Schultz PJ. Prevalence and risk factors for lameness in insulated free stall barns in Finland. Livest Scence. 2013;156:44-52.
113. Sjostrom K, Fall N, Blanco-Penedo I, Duval JE, Krieger M, Emanuelson U. Lameness prevalence and risk factors in organic dairy herds in four European countries. Livest Sci. 2018;208:44-50.
114. Sogstad AM, Fjeldaas T, Osteras O. Lameness and claw lesions of the Norwegian red dairy cattle housed in free stalls in relation to environment, parity and stage of lactation. Acta Vet Scand. 2005;46:203-17.
115. Solano L, Barkema HW, Pajor EA, Mason S, LeBlanc SJ, Heyerhoff JCZ, et al. Prevalence of lameness and associated risk factors in Canadian Holstein-Friesian cows housed in freestall barns. J Dairy Sci. 2015;98:6978-91.
116. Solano L, Barkema HW, Pajor EA, Mason S, LeBlanc SJ, Nash CGR, et al. Associations between lying behavior and lameness in Canadian Holstein-Friesian cows housed in freestall barns. J Dairy Sci. 2016;99:2086-101.
117. Sood P, Nanda AS. Lameness in crossbred cows: Prevalence, host level risk factors and reproductive performance. Ind J Anim Sci. 2013;83:379-82.
118. Sprecher, D. J., D. E. Hostetler, and J. B. Kaneene. A lameness scoring system that uses posture and gait to predict dairy cattle reproductive performance. Theriogenology 1997;47:1179-1187.
119. Stevančević M. Lameness at dairy cows, risk factors, prevention and control. Veterinarska Stanica. 2011;42:93-7.
120. Sun LP, Song YP, Riaz H, Yang LG. Effect of BoLA-DRB3 exon2 polymorphisms on lameness of Chinese Holstein cows. Mol Biol Rep. 2013;40:1081-6.
121. Tadich, N., E. Hettich, and G. van Schaik. Prevalence of lameness in cows from 50 dairy herds in southern Chile. Arch. Med. Vet. 2005;37:29-36.
122. Telezhenko E, Bergsten C. Influence of floor type on the locomotion of dairy cows. Appl Anim Behav Sci. 2005;93:183-97.
123. Thomas, H. J., G. G. Miguel-Pacheco, N. J. Bollard, S. C. Archer, N. J. Bell, C. Mason, O. J. Maxwell, J. G. Remnant, P. Sleeman, H. R. Whay, and J. N. Huxley. Evaluation of treatments for claw horn lesions in dairy cows in a randomized controlled trial. J. Dairy Sci. 2015;98:4477-4486.
124. Tisdall DA. The relationship between body condition score and mobility score in dairy cows on four commercial UK farms. Proceedings of the 17th International Symposium and 9th International Conference on Lameness in Ruminants, Bristol, UK, 11th-14th August 2013:275-6.
125. van Gastelen S, Westerlaan B, Houwers DJ, van Eerdenburg F. A study on cow comfort and risk for lameness and mastitis in relation to different types of bedding materials. J Dairy Sci. 2011;94:4878-88.
126. Vasseur, E., J. Gibbons, J. Rushen, D. Pellerin, E. Pajor, D. Lefebvre, and A. M. de Passille. An assessment tool to help producers improve cow comfort on their farms. J. Dairy Sci. 2015;98:698-708.
127. Vermunt J. Herd lameness - a review, major causal factors, and guidelines for prevention and control. Proceedings of the 13th International Symposium and 5th Conference on Lameness in Ruminants, Maribor, Slovenija, 11th-15th February 2004:3-18.
128. Vermunt JJ. The multifactorial nature of cattle lameness: a few more pieces of the jigsaw. Vet J. 2005;169:317-8.
129. Vosika B. Does robotic milking affect the claw condition and the occurrence of lameness in dairy cows? Proceedings of the 13th International Symposium and 5th

Conference on Lameness in Ruminants, Maribor, Slovenija, 11th-15th February 2004:199-200.
130. Weber A, Stamer E, Junge W, Thaller G. Genetic parameters for lameness and claw and leg diseases in dairy cows. J Dairy Sci. 2013;96:3310-8.
131. Wells SJ, Trent AM, Marsh WE, McGovern PG, Robinson RA. Individual cow risk factors for clinical lameness in lactating dairy cows. Prev Vet Med. 1993a;17:95-109.
132. Wells, S. J., A. M. Trent, W. E. Marsh, and R. A. Robinson. Prevalence and severity of lameness in lactating dairy cows in a sample of Minnesota and Wisconsin herds. J Am Vet Med Assoc. 1993b;202:78-82.
133. Wells SJ, Trent AM, Collier RJ, Cole WJ. Effect of long-term administration of a prolonged release formulation of bovine somatotropin (Sometribove) on clinical lameness in dairy cows. Am J Vet Res. 1995;56:992-6.
134. Wells SJ, Williamson NB, Robinson RA, Trent AM, Marsh WE. Some risk factors associated with clinical lameness in dairy herds in Minnesota and Wisconsin. Vet Rec. 1995;136:537-40.
135. Westin R, Vaughan A, de Passille AM, DeVries TJ, Pajor EA, Pellerin D, et al. Cow- and farm-level risk factors for lameness on dairy farms with automated milking systems. J Dairy Sci. 2016b;99:3732-43.
136. Winckler, C. and S. Willen. The reliability and repeatability of a lameness scoring system for use as an indicator of welfare in dairy cattle. Acta Agric Scand A Anim Sci. 2001;51:103-107.
137. Wongsanit J. Prevalence and risk factors for lameness in dairy cows raised in small holder farms in western Thailand. Kasetsart Veterinarians. 2015;25:47-55.
138. Yaylak E, Akbas Y, Kaya I, Uzmay C. The fffects of several cow and herd level factors on lameness in Holstein cows reared in Izmir Province of Turkey. Journal Anim Vet Adv. 2010;9:2714-22.
139. Zurbrigg K, Kelton D, Anderson N, Millman S. Tie-stall design and its relationship to lameness, injury, and cleanliness on 317 Ontario dairy farms. J Dairy Sci. 2005;88(9):3201-10.
